# Improvements and considerations for size distribution retrieval from small-angle scattering data by Monte Carlo methods

**DOI:** 10.1107/S0021889813001295

**Published:** 2013-02-14

**Authors:** Brian R. Pauw, Jan Skov Pedersen, Samuel Tardif, Masaki Takata, Bo B. Iversen

**Affiliations:** a Centre for Materials Crystallography, Department of Chemistry and iNANO, Aarhus University, DK-8000 Aarhus, Denmark; b Structural Materials Science Laboratory, RIKEN SPring-8 Centre, Hyogo 679-5148, Japan; c International Centre for Young Scientists, National Institute of Materials Science, Tsukuba 305-0047, Japan; d Department of Chemistry and iNANO, Aarhus University, DK-8000 Aarhus, Denmark; e Quantum Order Research Group, RIKEN SPring-8 Centre, Hyogo 679-5148, Japan

**Keywords:** structure analysis, small-angle scattering, Monte Carlo methods, particle size distribution

## Abstract

Monte Carlo (MC) methods, based on random updates and the trial-and-error principle, are well suited to retrieve form-free particle size distributions from small-angle scattering patterns of non-interacting low-concentration scatterers such as particles in solution or precipitates in metals. Improvements are presented to existing MC methods, such as a non-ambiguous convergence criterion, nonlinear scaling of contributions to match their observability in a scattering measurement, and a method for estimating the minimum visibility threshold and uncertainties on the resulting size distributions.

## Abstract 2

A method is presented and applied, capable of retrieving form-free particle size distributions complete with uncertainties from small-angle scattering patterns. Special attention is paid to particle observability in the scattering patterns, accurate estimation of data uncertainty and the effect of uncertainty on the resulting size distribution statistics.

## 1. Introduction

The search for generally applicable methods capable of determining structural parameters from small-angle scattering patterns for a broad range of samples has yielded several viable methods. For monodisperse systems consisting of identical particles, these methods attempt to find a form-free solution to the pair-distance distribution function, whereas for polydisperse systems the aim is to determine the particle size distribution. In both cases, relevant transformation should yield the observed scattering pattern (Krauthäuser *et al.*, 1996 ▶).

There are indirect transform methods (ITMs) based on regularization techniques, which impose that the solution is as smooth as possible (Glatter, 1977 ▶, 1979 ▶; Moore, 1980 ▶; Svergun, 1991 ▶; Pedersen, 1994 ▶), and Bayesian and maximum entropy ITMs, which find the most likely solution using a Bayesian approach and entropy maximization, respectively (Hansen, 2000 ▶; Hansen & Pedersen, 1991 ▶). There are also methods available based on Titchmarsh transforms for determining size distributions (Botet & Cabane, 2012 ▶; Mulato & Chambouleyron, 1996 ▶; Fedorova & Schmidt, 1978 ▶).

Another class of methods, such as the structure interference method (SIM; Krauthäuser *et al.*, 1996 ▶) and some Monte Carlo (MC) methods (Martelli & Di Nunzio, 2002 ▶; Di Nunzio *et al.*, 2004 ▶), assume a particular shape and do not appear to require smoothness constraints. These only have a positivity constraint and have so far been limited to size distributions of sphere-shaped scatterers. These methods can be used to extract the particle size distribution function of systems of scatterers whose shape is known or assumed, and not affected by concentration effects (Krauthäuser *et al.*, 1996 ▶; Martelli & Di Nunzio, 2002 ▶; Di Nunzio *et al.*, 2004 ▶). The MC variant approaches the optimization by trial and error, whereas the SIM uses a conjugate gradient approach (Krauthauser, 1994 ▶). Both are conceptually easier than the ITMs and methods based on Titchmarsh transforms and provide stable and unique solutions (Martelli & Di Nunzio, 2002 ▶; Di Nunzio *et al.*, 2004 ▶; Krauthäuser *et al.*, 1996 ▶).

Upon implementation of one such method by Martelli & Di Nunzio (2002 ▶), hereafter referred to as ‘the Martelli method’, several noteworthy changes were made to that method, which are presented here. We briefly reiterate the working principle and highlight the differences of the presented MC method from the Martelli method. Then, a general solution for detection limits is derived for particles in a polydisperse set. This aids the MC method as it allows for improved contribution scaling during the optimization procedure and indicates detectability limits in the final result. Lastly, a convergence criterion is defined for the MC method, allowing for the calculation of uncertainties in the resulting size distribution. This method is shown to be applicable to scattering data obtained during the synthesis of AlOOH nanoparticles.

## 2. A brief overview of the implemented method

The MC method proposed here is essentially identical to that by Martelli & Di Nunzio (2002 ▶). The differences can be summarized as follows: (1) we apply a help variable 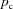, which is used in the optimization to (partially) compensate for the effect of size on the scaling of the form-factor scattering, whose magnitude is derived through the definition of ‘maximum observability’; (2) a limited fixed number of sphere contributions is used and constantly replaced, this number being determined from the optimization speed; (3) a strict convergence criterion is defined on the basis of the estimated data uncertainty; and (4) standard deviations are calculated on the result through repetitive application of the MC method. A summary of the MC method is given below.

### 2.1. Step 0: determination of the feature size limits

If not explicitly defined, a reasonable estimate has to be made for the radius range of the determinable features in the scattering pattern. This estimate is based on the measured data set, in particular the minimum measured 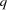 and the spacing between subsequent data points in 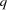. Here, 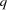 is defined as 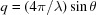, with λ the wavelength of the radiation and 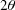 the scattering angle. The radius of the maximum detectable feature 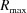 in a small-angle scattering measurement is mostly defined by the size of the direct beam spot on the detector. As this is often wholly (but just) obscured by the beamstop, a reasonable assumption for 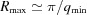, where 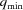 is the smallest measured value of 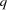 in the corrected data set. If, however, the beamstop is oversized compared to the actual beam size, 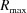 may be more closely related to the spacing between 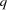 data points, so that 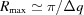, where 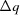 is the smallest distance between two neighbouring 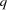 points in the data set.

Similarly, a reasonable estimate for the minimum radius of distinguishable features 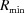 can be defined. As above, this can be related to the largest available 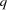 value in the data points of the measured data set. It thus can assume the value of 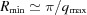, where 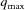 is the largest measured value of 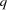 in the corrected data set. Any value for 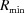 smaller than about 0.3 nm, however, would define features approaching the length of chemical bonds, where the commonly used assumption of uniform electron density no longer applies. In the interest of generality in the MC method, 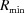 is set to zero, but it is of importance to consider these fundamental limitations when interpreting features in the result.

If no *a priori* information on the bounds is supplied, the radius range is thus set to 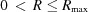, where 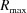 is the largest of the two aforementioned alternatives.

### 2.2. Step 1: preparation of the procedure

The initial guess of the total scattering cross section is calculated for a fixed number of contributions 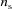 (typically 200–300) from spheres whose radii 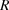 are randomly sampled from a bound uniform distribution (bound by the radius range described in the previous section). These radii can therefore assume any value within the size bounds. This initial guess is calculated using the general equation
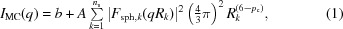
where the sum runs over all spheres in the finite set and 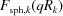 is the Rayleigh form factor for sphere 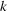, normalized to 1 for 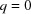. 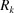 is the radius for sphere 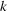. 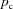 is a parameter adjustable in the range 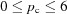, biasing the volume weighting of the contributions, and will be discussed in detail hereafter. 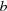 is a constant background term, and 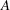 is a scaling factor, which is related to the volume fraction ϕ of the scatterers through
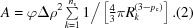
Here the sum runs over all spheres in the finite set and 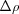 is the scattering contrast. The volume fraction of the scatterers ϕ is defined as
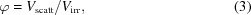
where 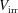 is the irradiated sample volume and 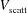 is the total scatterer volume in 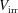.

Given the limited (fixed) number of spheres used for the calculation of the scattering cross section, a large sphere could easily dominate if normal volume-squared scaling is used for each sphere contribution (*i.e.* 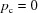). As an example, a sphere of 10 nm would require a million spheres of 1 nm for an equivalent contribution (at 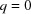). It is evident, therefore, that describing the scattering from polydisperse sets of spheres would require very large numbers of smaller sphere contributions to have any distinguishable impact on the scattering on top of that originating from a few large scatterers. The factor 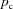 in equation (1) is thus used as a computational aid to partially suppress this effect of the volume-squared sphere contribution scaling, by increasing the contribution from the smaller spheres in the set. The recommended value for 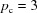, but in practice it can be freely varied in the range 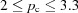 as shown in §[Sec sec2.5]2.5.

The choice of 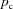 only affects the speed of the calculation and does not affect the result as it is compensated for in 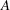 [equation (2)] and therefore also when visualizing the results (it can, as will be discussed later, also slightly affect the standard deviation of the result when set to extreme values, as shown in the supplementary information^1^). Setting 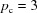 results in the internal calculation using volume-weighted spheres (as opposed to volume-squared-weighted spheres for 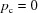), and setting 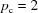 makes each sphere in the MC procedure assume an identical ‘observability’, which is a proposed measure for the maximum visibility of a scatterer in a polydisperse system, defined and explored in §[Sec sec3]3. Lastly, 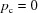 removes this compensation altogether from the procedure; this approach is suitable only for very narrow distributions owing to the limited number of spheres used in the MC calculation.


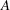
 and 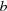 are tuned to the measured scattering through optimization using a least-squares residual minimization procedure, minimizing the reduced chi squared 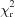 (Pedersen, 1997 ▶):
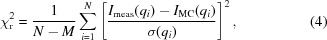
where 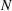 denotes the number of data points and 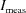 and 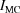 the measured and calculated model scattering cross section, respectively. 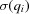 is the estimated error on each measured data point, whose estimation method is detailed in §[Sec sec4]4. 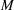 is the number of degrees of freedom in the fitting model, but is unfortunately ill-defined in an MC model and is thus set equal to 2 to account for the scaling parameter 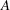 and background contribution parameter 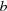.

### 2.3. Step 2: optimization cycle

After random selection of the 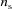 sphere radii and the subsequent calculation of the initial guess, the Monte Carlo optimization cycle then begins. A random sphere is selected from the set of 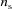 spheres, and a change of its radius to another random value within the previously discussed radius bounds is suggested. The intensity after this suggested change is calculated by subtracting the contribution of the previous sphere radius and adding the contribution of the new sphere radius, and re-optimizing the scaling factor 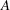 and background level 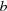. The suggested radius change is accepted if it improves the agreement between measured and MC intensity, *i.e.* if the change reduces the 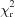 value. Similar to the SIM and the Martelli method, a rejection–acceptance mechanism that occasionally accepts ‘bad moves’ was found not to be necessary (Krauthäuser *et al.*, 1996 ▶; Martelli & Di Nunzio, 2002 ▶; Di Nunzio *et al.*, 2004 ▶).

This method differs from the Martelli method in that the Martelli method continually attempts to add new sphere contributions to an ever growing set, leaving the prior established set of contributions untouched. Conversely, the adaptation presented here leaves the number of sphere contributions in the set unchanged and repeatedly tries to change the radius of a random contribution in the set. An attempt was made to adjust the MC method here to add spheres instead of changing sphere radii, more closely following the method presented by Martelli. It was found, however, that such a modification would occasionally prevent convergence.

### 2.4. Step 3: convergence and post-optimization procedures

The optimization is ceased once the condition 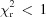 has been reached (*cf.* §[Sec sec4]4). If convergence has not been reached within a certain number of optimization cycles (here set to one million), and fails to do so repeatedly, the pattern is considered unsuitable for fitting with this method.

After convergence has been reached, the partial volume fraction for a sphere contribution 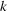, 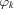, is then calculated through reformulation of equation (2):
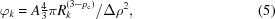
where *A* is known through least-squares fitting from step 2. If this equation is calculated for all 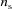 sphere contributions, the volume fraction of the scatterers ϕ can be calculated as 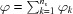.

For visualization and analysis purposes, the obtained partial volume fractions 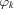 can be distributed over a number of radius bins to form a histogram or a volume-weighted size distribution (an example of which is given in §[Sec sec7]7). In this histogram, each radius bin (denoted with the subscript *R*bin) is defined by its bin edges, 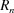 and 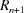, also known as the class limits. If a particular sphere contribution radius falls within the bin, its volume fraction 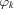 is added to the contents of the bin. In other words, each radius bin defined by its edges 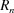 and 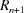 will assume the value that is the sum of all partial volume fractions of contributions whose radii fall within that bin to make up the bin volume fraction 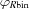. If instead of the volume-weighted size distribution a number-weighted size distribution is sought, this procedure is identical except that each contribution adds 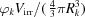 to the bin.

Accurate mean and standard deviations for the final volume-weighted size distribution are calculated from the results of several tens of independent repetitions of the whole MC procedure. This is considered to provide a reasonable check for ambiguity, and such standard deviations may aid in the comparison of the size distribution obtained with small-angle scattering with those obtained using other methods.

### 2.5. Determination of the number of contributions *n*_s_ and compensation parameter *p*_c_

A suitable value must be found for the number of contributions 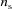 in the MC method. Too large a number of contributions will result in excessive computation time and may effect an overly smooth solution and underestimated standard deviations on the result (an effect shown in the supplementary information). Too few contributions will also lead to a long computation time as the few contributions need to assume increasingly strict radii. Furthermore, the number of required contributions is dependent on the data quality, with data of higher quality requiring more contributions to achieve convergence.

**Figure 1:**
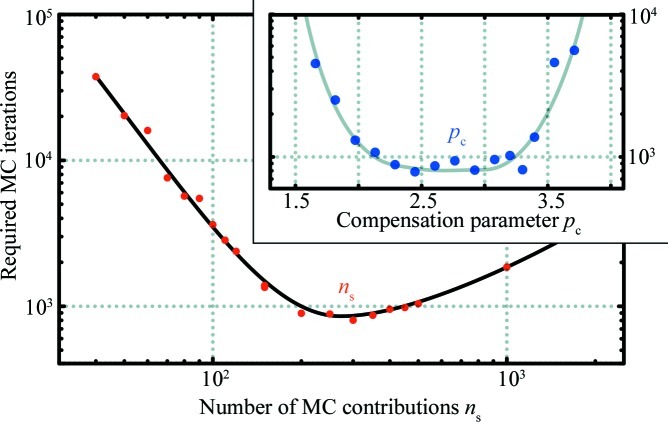
Optimization of the number of MC contributions 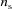 (main figure) and 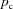 (upper right, calculated for 300 contributions) for the Boehmite example data (see §[Sec sec6]6) by selecting the values requiring the least number of steps. Lines are added to guide the eye.

It is therefore suggested that 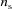 is set so that convergence is achieved in as few MC iterations as possible, *i.e.* an efficiency-maximizing approach. Plotting the number of iterations required for convergence *versus* 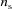 will show what value should be used for a particular data set (Fig. 1 ▶). For consistency in series of measurements, such as time or temperature series, it is recommended to fix 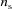 to a single value suitable for the majority of the measurements.

A similar approach can be used to fine-tune 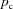. As shown in Fig. 1 ▶, however, there are a wide range of values with nearly identical calculation efficiencies. This indicates that suitable choices for this example lie within the range 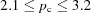.

## 3. Observability of isolated spheres in a polydisperse set

As the scattering power of particles scales proportionally to their volume squared, the scattering of smaller particles in a polydisperse set is quickly drowned out by the signal of the large particles. This effect, however, is partially compensated for by the different *q* dependence of the scattering of the smaller particles.

To investigate how large this compensatory effect is, we define the ‘maximum observability’ Obs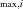 of a particle 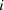 in a set 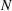 as the maximum of the ratio of its individual contribution 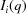 to the total intensity 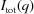 in the range [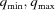] [equation (6)]. The location of the maximum is denoted as 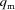.
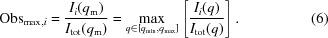
The individual contribution 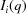 of sphere 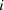 with radius *R_i_* is given by equation (1) with 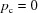.

**Figure 2:**
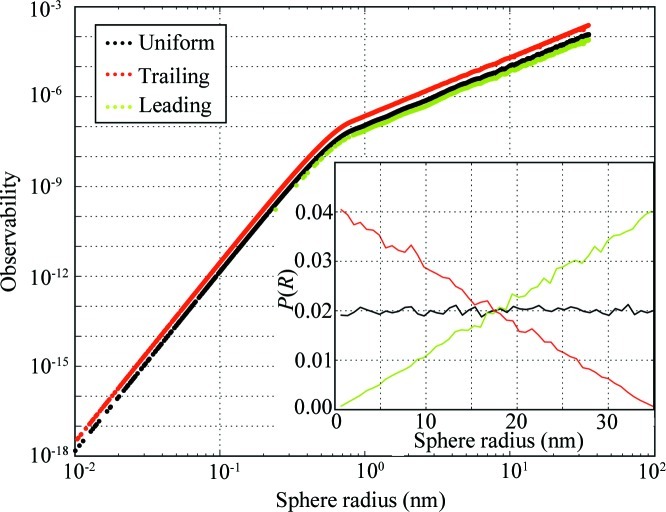
Observability for three unimodal number-weighted size distributions sampled using 50 000 spheres within 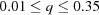. The number frequency plot of the size distributions is shown in the inset, with the distributions divided over 50 radius bins and normalized to 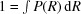. A change in slope between 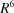 and 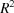 is observed for all three distributions at 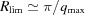.

The maximum observability has been calculated for three separate unimodal distributions, with each number-weighted size distribution *P*(*R*) sampled using 50 000 sphere radii in the range 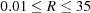
 nm. There are three distributions: (1) a uniform distribution, (2) a ‘trailing’ triangular distribution with its mode set to 0.01 nm and (3) a ‘leading’ triangular distribution with its mode set to 35 nm (as shown in the inset of Fig. 2 ▶). Fig. 2 ▶ shows the maximum observability to be unaffected by the choice of distribution. From the figure, it is evident that the maximum observability scales as 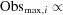 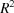 for particles with sizes larger than 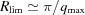. Particles smaller than 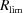 exhibit an observability scaling in line with the volume-squared intensity scaling (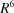). A similar, independently determined lower limit has been obtained through MC size distribution retrieval exercises (available in the supplementary material).

The information on the observability can be used for three purposes. First and foremost, there is a clear indication of the limits of small-angle scattering for resolving the smaller sizes, which can be linked to the maximum measured 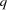. This is evident from Fig. 2 ▶ as observability scales with radius to the sixth power for particles with radii smaller than 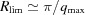, which renders their contribution rapidly indistinguishable. This reinforces the point that the minimum detectable feature radius 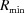 can be estimated to be 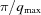, as discussed in §[Sec sec2]2.

Secondly, this information directly gives us a value of 2 for 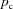, which will result in an equal maximum observability for every contribution in the MC method, provided that the contribution radii fall within the bounds imposed by the angular limits of the measurement. It is still considered preferable, however, to tune 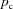 for each sample (series) according to the method described in §[Sec sec2.5]2.5.

Thirdly, the concept of observability can be used to determine whether a particular feature in a size distribution is significant or not. To be more precise, we can calculate what level is required in any radius bin (*cf.* step 3 of §[Sec sec2]2) in order to contribute scattering that can be distinguished in the fitted scattering pattern above the scattering pattern uncertainty 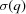. In the MC calculation, a model component 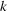 contributes scattering 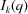 which is proportional to its partial volume fraction 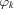 [where 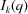 is calculated using equation (1) for a single contribution and with 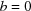]. Its scattering contribution can be considered to become distinguishable at a minimum partial volume fraction 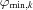 where it contributes at least as much as the data uncertainty:
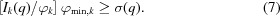
This allows the calculation of 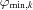 in two ways: (1) as the minimum of all values calculated over 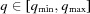 or (2) as the value at 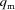, where the contribution from 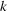 is strongest [equation (6)], which in practice turns out to be a good approximation for (1):
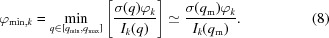
For a given radius bin in the visualization (as defined in step 3 of §[Sec sec2]2), we can then estimate the minimum visibility threshold 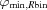 of that bin. This can be estimated as either the average of the 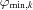 values of the components that fall into this bin or – to be on the safe side – the largest value of 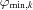 in that bin. Likewise, if the visualization choice was of a number-weighted distribution following the procedure described above, the minimum number required to make a measurable impact can be calculated from the minimum 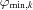 through 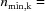 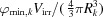.

In this way, any size distribution visualization (*e.g.* number-weighted or volume-weighted size distributions) can contain a line indicating a rough estimate for the minimum detectability threshold (an example is shown in §[Sec sec7]7). Inclusion of this line can prevent the drawing of erroneous conclusions.

## 4. Data point weighting and convergence criterion

In most small-angle scattering measurements, the many data points 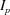 collected from each pixel 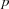 on the detector are reduced into a small number of 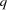 bins, 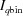, before the data analysis procedures. In this reduction step, each measured data point collected between the bin edges 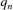 and 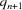 is averaged and assumed valid for the mean 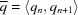, in a process known colloquially as ‘radial integration’ or ‘radial averaging’. In other words, 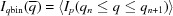. Keeping track of the uncertainties during this process (and other steps in the data correction and reduction methodology) will result in the availability of uncertainties on the binned intensity data points.

These estimates of the level of uncertainty or ‘errors’ on each given data point are invaluable in assessing the veracity of model fitting results: to determine whether the analysis provided a solution to within the uncertainty estimate. Additionally, knowledge of the uncertainty can help unlink the model fitting result from more arbitrary parameters such as the number or spacing of the integration bin edges. By weighting of the goodness-of-fit parameter used in the least-squares minimization function by this error [*cf.* equation (4)] uncertainties on the MC solution can be established.

The counting-statistics-based Poisson error 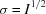, where 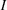 is the number of detected counts in the raw data, gives the lower limit of the uncertainty estimate. These errors need to be appropriately propagated through the binning procedure and various data corrections such as dead time, dark current and background. Furthermore, if this estimate is exceeded by the sample standard error of the mean of the values contained in each individual 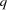 bin, the sample standard error should be the preferred error estimate for that bin as it can account for some detector irregularities. This sample standard error for each bin 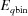 is defined as
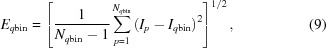
where 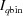 is the mean intensity in the 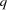 bin, 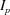 the intensity of data point 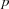 in the bin and 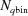 the total number of data points in the bin.

Lastly, it is commonly challenging to get the absolute uncertainty of the measured intensity below 1%, even with the most elaborate data corrections in place, as shown by Hura *et al.* (2000 ▶), who achieved an overall uncertainty of about 2%. Thus, the uncertainty should be taken to be 1% of the measured intensity if this value exceeds the other two estimates. These errors can then be used in equation (4) to determine the goodness-of-fit parameter for an MC proposition.

The advantage of using these errors in the expression for 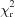 is that, if this parameter drops below one, the deviations between model and measured intensities are on average smaller than the statistical uncertainties. This thus provides a cutoff criterion for the MC method, allowing for the estimation of the mean and standard deviations of the final particle size histogram (as shown in §[Sec sec7]7). Additionally, by using these errors in the expression for the goodness-of-fit parameter, the intensities are weighted by their relative errors in the fitting procedures and thus become less sensitive to arbitrary values such as bin widths or number of data points used in the fit.

Since the MC method does not provide us with an intensity at the same level as the measured intensity, and as there often is a constant background associated with small-angle scattering patterns as described by Ruland (1971 ▶) and Koberstein *et al.* (1980 ▶), these two parameters will have to be determined separately. Thus, after every MC proposed change, but before calculation of the goodness of fit, an intermediate least-squares minimization routine is applied to optimize the model intensity scaling 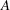 and background parameter 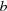 [equation (1)]. As 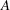 does not affect the shape of the scattering pattern, the MC result remains unaffected. The addition of the background term may affect the presence of MC contributions with an effectively flat scattering profile, whose radii will be much below the radius limit imposed by the maximum measured *q*. If required, the least-squares minimization method may be expanded to include more terms, at the cost of speed and stability. One example of such an inclusion could be to include a power-law slope (with optional cutoff) to compensate for scattering from large structures or some interparticle scattering effects (Pedersen, 1994 ▶; Beaucage, 1995 ▶, 1996 ▶).

## 5. Uncertainties on the resulting distribution

One common criticism of MC methods is the potential for ambiguity in the result, *i.e.* the possibility to arrive at any number of equally valid but unrelated solutions. This MC implementation addresses that issue in two ways, firstly by limiting the number of sphere contributions through association with data quality, and secondly through determination of the variance between several tens of independent solutions, each time optimizing until 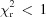 has been achieved. As it is difficult to compare and visualize sets of radii, each resulting set is converted using a histogramming procedure to a density map or a volume-weighted size distribution. The variance between the histograms of the independent solutions will provide a mean and standard deviation for each histogram bin.

The visualization of the resulting data set in a histogram can be fine-tuned to some degree by adjustment of the number and spacing of the histogram radius bins. A greater number of radius bins (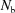) will result in more detail at the cost of larger standard deviations on the bin values. A good estimate for 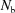 can be found by means of the sampling theorem 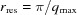, 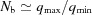, assuming the largest measurable dimension is identical to the measurement limit (Hansen & Pedersen, 1991 ▶; Moore, 1980 ▶; Taupin & Luzzati, 1982 ▶). However, 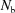 also has to be dependent on the uncertainty of the underlying data set: high-quality data with small uncertainties will result in smaller standard deviations on the visualized result, indicating that 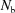 can be doubled for more detail. A lot of noise or a large uncertainty in the measured data, on the other hand, will result in a large standard deviation on the histogram, indicating that 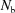 should be halved. While none of this affects the result of the MC optimization, the balance between detail and accuracy of the result visualization cannot be strictly defined. Lastly, the spacing between the bin edges can be adjusted in the visualization, which can be necessary if the result spans several decades in size (for which logarithmically spaced bin edges can be applied) or has sharp features [for which a natural binning has been suggested by Ilavsky & Jemian (2009 ▶)]. It is important to emphasize that none of these adjustments to the spacing or number of bins affect the MC result, and that there is an inherent link between the standard deviation and the choice of visualization parameters.

It should be noted, however, that while the standard deviations on the histogram are linked to the data uncertainty, there remains room for improvement as the standard deviation (but not the mean) on the resulting histogram can be affected slightly by the choice of 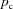 (especially when set to 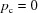 or 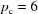, as shown in the supplementary information). Despite this, the procedure does provide the user with a reasonable estimate of the standard deviation, which can be coupled with the observability information to obtain a size distribution with statistics.

## 6. Experimental

### 6.1. Synthesis

Boehmite (AlOOH) particles were synthesized *in situ* using an automated and modified version of a high-pressure high-temperature reactor (Becker *et al.*, 2010 ▶). The sapphire capillary in which the reaction takes place has an inner diameter of 1.0 mm and an outer diameter of 1.57 mm. The particles were synthesized from a solution of 0.5 *M* Al(NO_3_)_3_ precursor in water. The start of the reaction was considered to be the moment at which the pressurized solution [maintained at a pressure of 250 bar (25 MPa)] is heated to its reaction temperature of 548 K. The measurement used in this paper was obtained 1700 s from the start of the reaction. Further details and results will be presented in a forthcoming paper.

### 6.2. Beamline details

Synchrotron SAXS experiments were performed at the BL45XU beamline of the SPring-8 synchrotron in Japan. The beam was collimated to a 0.4 × 0.2 mm beam (horizontal by vertical, respectively), with photons with a wavelength of 0.09 nm. The sample-to-detector distance was 2.59 m. The scattering patterns were recorded on a Pilatus 300k detector whose total surface area covers 33.5 × 254 mm, consisting of 195 × 1475 pixels measuring 0.172 × 0.172 mm. Transmission values were determined using in-line ionization chambers. The polarization factor was assumed to be 0.95. The measurements were collected at a rate of 1 Hz.

### 6.3. Data correction

The data were corrected for background (water at 548 K and 250 bar), incoming flux, measurement time, transmission, polarization and spherical correction and calibrated to absolute units using a glassy carbon sample from series H, supplied by Dr J. Ilavsky from the Advanced Photon Source (Zhang *et al.*, 2009 ▶). Statistics were calculated according to the procedure outlined in §[Sec sec4]4, with the minimum possible error set to 1% of the measured intensity. The scattering contrast Δρ^2^ is set to 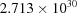
 m^−4^.

## 7. Results and discussion

**Figure 3:**
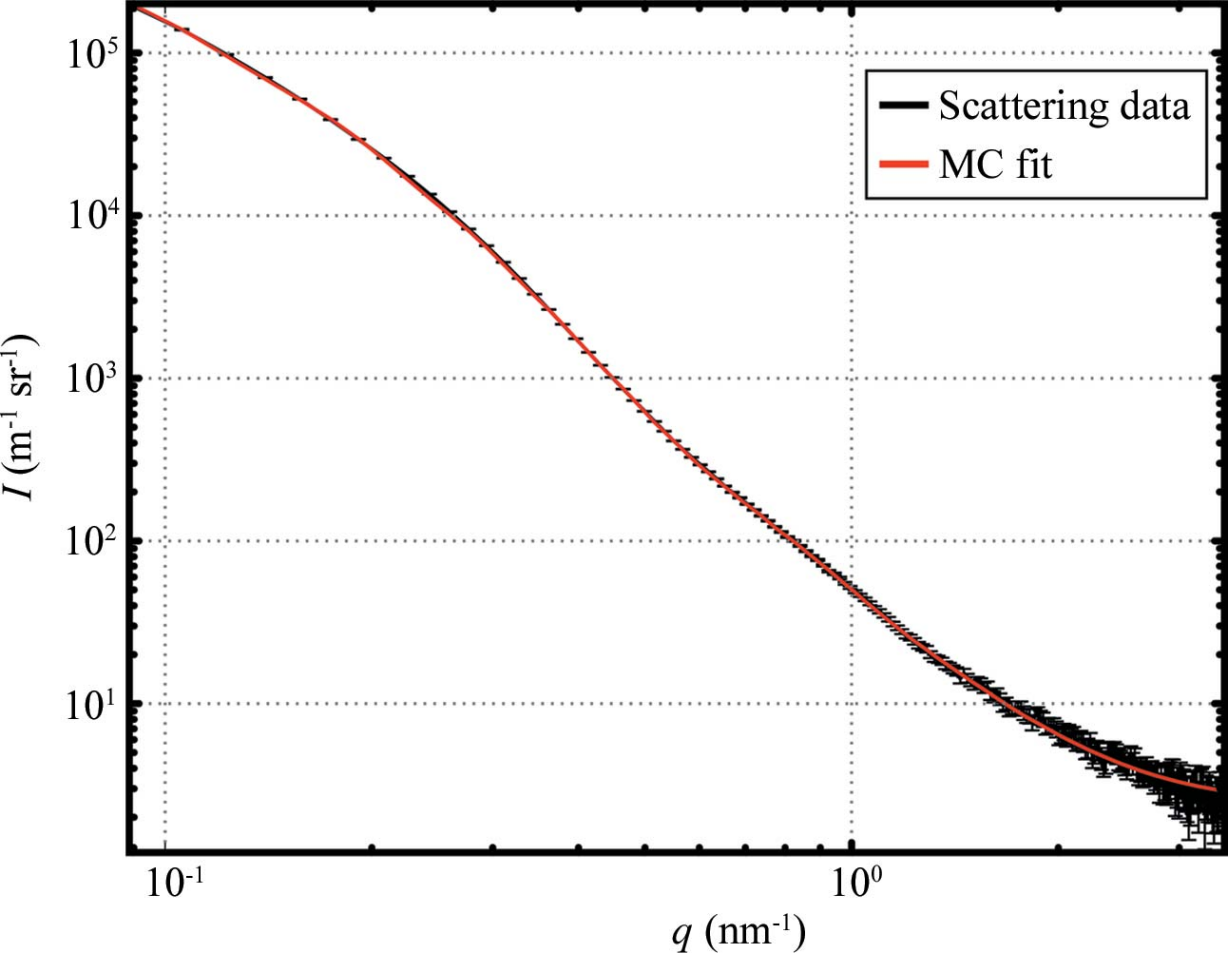
Data (black) and MC fit [grey (red in the electronic version of the journal), using 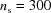 and 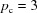] for a 1 s measurement in a time series of AlOOH nanoparticles in aqueous solution. The MC fit is at convergence. The background value resulting from the optimization is 2.4 (2) m^−1^ sr^−1^.

The collected data set, consisting of 200 data points, the errors and the MC fit are shown in Fig. 3 ▶, where the MC fit intensity is the average of 100 repetitions of the MC procedure. While not as fast in its current implementation as some of the alternative ITMs, the calculation of all 100 repetitions takes about 30 s on a single core of an Intel Core i7 processor running at a clock speed of 1.8 GHz. This performance can be improved by an estimated factor of 50 through compilation of selected parts of the code, as well as exploitation of multicore processing. Given that the speed is not yet a limiting factor in the overall methodology, no steps have been made in this direction.

**Figure 4:**
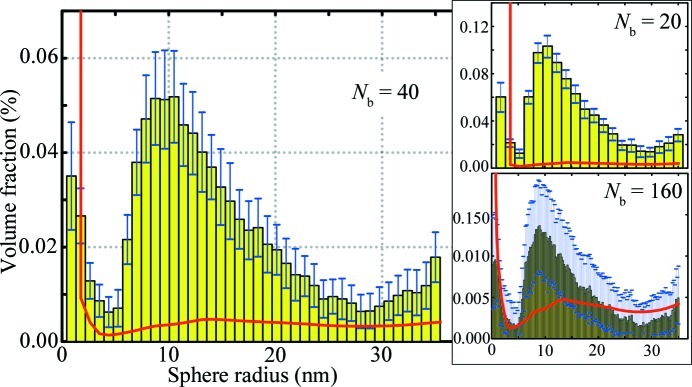
Volume-weighted size distribution as used for the MC fit shown in Fig. 3 ▶, for 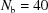. Error bars indicate sample standard deviation over 100 repetitions. The minimum visibility threshold 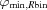 is shown as a thick grey (red in the electronic version of the journal) line. The right-hand figures show the effect of selecting 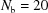 and 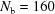, with a clear effect on 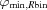 as well as the uncertainty estimates.

While a single run also delivers a model intensity on average to within the determined error, the mean intensity is shown here as it matches the mean of the size distributions shown in Fig. 4 ▶. The error bars indicate the standard deviation of the histogrammed values for each of 100 repetitions. This figure also contains the estimate of the minimum visibility threshold 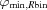.

The volume-weighted size distribution shows a distinctly triangular size distribution between 5 and 30 nm, with the distribution maximum at 10 nm. The total volume fraction of measurable precipitates is 0.88%, which is close to, but not quite, the 0.99% expected for full conversion of the precursor. This deviation may be attributed to large uncertainties on some of the values used in the data reduction (*e.g.* transmission factor, capillary diameter), partial precipitation of the nanoparticles at the bottom of the capillary or even precipitates growing beyond the coherence limit (which then no longer contribute to the scattering pattern).

In this example, the number of histogram bins is 40, the recommended number of divisions dictated by the sampling theorem, but one can choose more or fewer bins. The effect of this is shown in the subplots in Fig. 4 ▶ for 20 and 160 histogram bins, which clearly show the relation between the standard deviation in the bins and the minimum visibility threshold 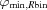. If the number of histogram bins is high, the uncertainty for each value is equally large and the minimum visibility threshold increases. If the number of bins is reduced, both the uncertainty and 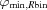 reduce, at the cost of a loss of detail.

## 8. A final comment

All the above results were obtained assuming that the scatterers are spherical in shape. This does not have to be the correct particle shape for the method to arrive at a solution, as the size distribution and shape cannot both be uniquely distinguished from scattering patterns alone (which has been tested for simulated isotropic scattering patterns from polydisperse sets of prolate and oblate ellipsoids, as shown in the supporting information). The solution from the MC method, then, shows the user what the size distribution would be *if* the scattering pattern originated from spherical particles.

If the shape of the scatterers is known from other investigations such as electron microscopy, and deviates from a spherical shape, this information can be used to obtain the correct size distribution for that particular shape (Pedersen *et al.*, 1996 ▶). This can be done either by adjusting the particular scattering function in the MC method [as recently shown for rod-like precipitates in MgZn alloys by Rosalie & Pauw (2012 ▶)] or by analysis of the shape-independent correlation function 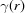, which can be calculated from the sphere-based MC method result (Feigin & Svergun, 1987 ▶).

## 9. Conclusions

Discussed in this paper are modifications to the Martelli MC method, the general veracity of the result and the application of it to a SAXS measurement. It is shown that by using the methodology described in this paper a particle size distribution can be retrieved from a scattering pattern, uncertainties can be estimated for the particle size distribution and the minimum number of particles required to make a measurable impact on the scattering pattern (the minimum visibility threshold) can be indicated for each feature in the distribution.

The MC code is available for inspection, improvements and application under a Creative Commons Attribution-Share­Alike license and the latest copy will be freely supplied by the author upon request.

## Supplementary Material

Supplementary material file. DOI: 10.1107/S0021889813001295/ce5145sup1.pdf

## Footnotes

1 Supplementary material is available from the IUCr electronic archives (Reference: CE5145). Services for accessing this material are described at the back of the journal.
