# Supplementary material for: Improvements and considerations for size distribution retrieval from small-angle scattering data by Monte Carlo methods
Source: J Appl Crystallogr. 2013 Feb 14;46(Pt 2):365–71. doi: 10.1107/S0021889813001295 (PMC3627408; doi:10.1107/S0021889813001295)
Supplement: Supplementary file 1 [file j-46-00365-sup1.pdf]

A showcase of the Monte-Carlo method.

Supplementary information to: “Improvements and considerations for size distribution retrieval from small-angle scattering data by Monte-Carlo methods”

brian@stack.nl

Brian R. Pauw,[a,b] Jan-Skov Pedersen,[c] Samuel Tardif,[b] Masaki Takata[b] and Bo B. Iversen[a]

[a] *Center for Materials Crystallography, Department of Chemistry and iNANO, Aarhus University, DK-8000 Aarhus, Denmark,* [b] *RIKEN SPring-8 Center, Sayo, Hyogo, 679-5148, and* [c] *Department of Chemistry and iNANO, Aarhus University, DK-8000 Aarhus, Denmark*

(Received 0 XXXXXXX 0000; accepted XXXXXXX 0000)

Abstract

This supplementary information explores some details of the Monte-Carlo fitting procedure by application to simulated data. This is not meant to be an exhaustive exploration (if such an exploration is even possible), but covers several common types of data and model systems.

1. Definitions

Length units are given in nanometers (unless otherwise stated).

2. Simulation settings

Unless otherwise stated, the following settings are applied:

- Simulated data spans a q-range of 0.1 to 3.5 inverse nanometer unless otherwise stated. This range is (in the authors experience) a commonly accessible range on SAXS instruments. The q-range is described using 100 linearly-spaced points.
- Size distributions are limited to the range defined by $R_{min} = \pi/q_{max}$ and $R_{max} = \pi/q_{min}$, approximately from 0.9 nm to 31.4 nm.
- All size distributions calculated are volume-weighted size distributions unless otherwise stated.
- The maximum intensity is normalised to 1 million “counts”, and rounded to discrete values to more closely approximate a real measurement
- The uncertainty on the intensity (+/- one standard deviation) is estimated to be the maximum of either 1% of the intensity value or the square root of the number of “counts”.
- The Monte-Carlo fit is optimised 100 times to $\chi_r^2 < 1$, the plotted intensity is the mean of the result, and the size distribution is shown as the mean +/- one standard deviation of all 100 optimisations.
- The volume-weighted size distributions are vertically normalised to a total volume fraction of 1. In real systems, provided correct absolute units, transmissions, thickness, etc., this would indicate the actual volume fraction of precipitates in the sample.
- For the generation of the scattering patterns, the distributions are randomly sampled using a total of 10000 spheres.
- If randomly sampled radii are negative, they are not used.
- The MC method is applied with 200 spheres for speed. This does exaggerate the standard deviation on the resulting histograms due to the numerical noise of the

quantisation of each MC solution.

Python (version 2.7.2) code to generate the distributions is provided.

3. Unimodal distributions

3.1. Monodisperse spheres

A monodisperse set of spheres of 10 nm is simulated. The MC method has no problem retrieving this distribution (Figure 1). This retrieval takes a bit longer as there is only a very limited range of valid sizes for the spheres to end up in, and the chance of hitting that range randomly is rather small.

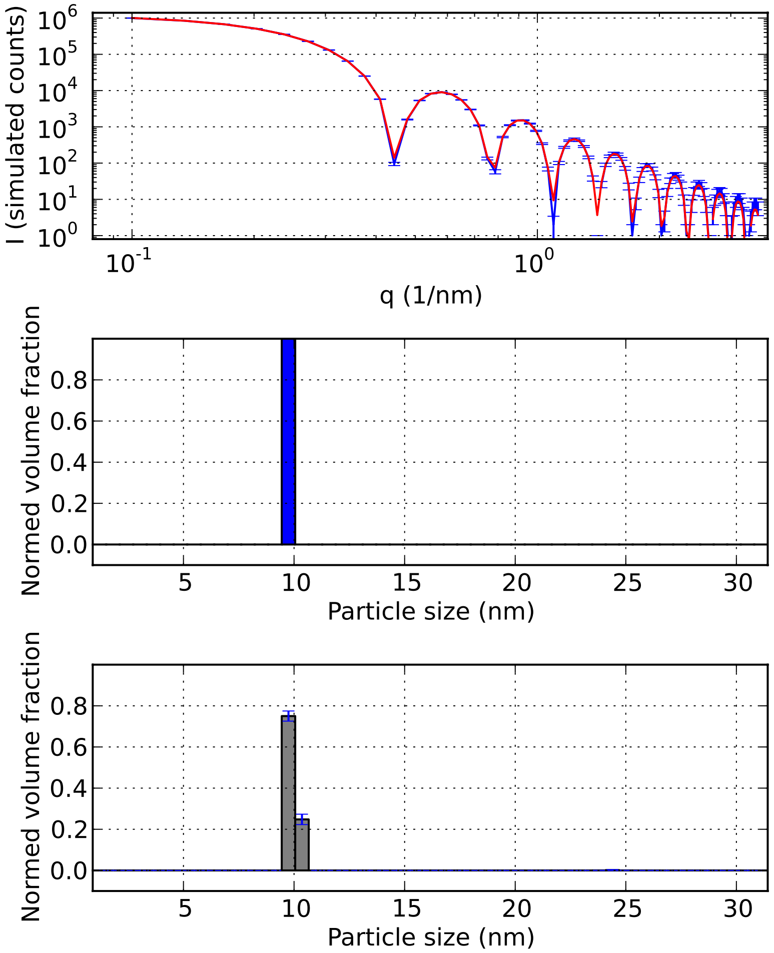

Fig. 1. Top: Simulated intensity (blue) and MC fit (red) of a monodisperse distribution of spheres with a mean of 10 nm (middle). Bottom: Retrieved size distribution.

3.2. Narrow normal distribution

A normal distribution with the mean at 10 nm and a standard deviation of 2 nm is simulated. The MC method has no problem retrieving this distribution (Figure 2). It has furthermore been computed using two more (extreme) values for p_c: 6 and 0 (Figures 3 and 4, respectively). The choice of p_c is shown not to affect the mean result but does affect the standard deviations due to increased sensitivity to numerical noise in the small- and large sizes, respectively. The effect of numerical noise can be reduced

by increasing the number of sphere contributions in the MC calculation at the cost of computing time.

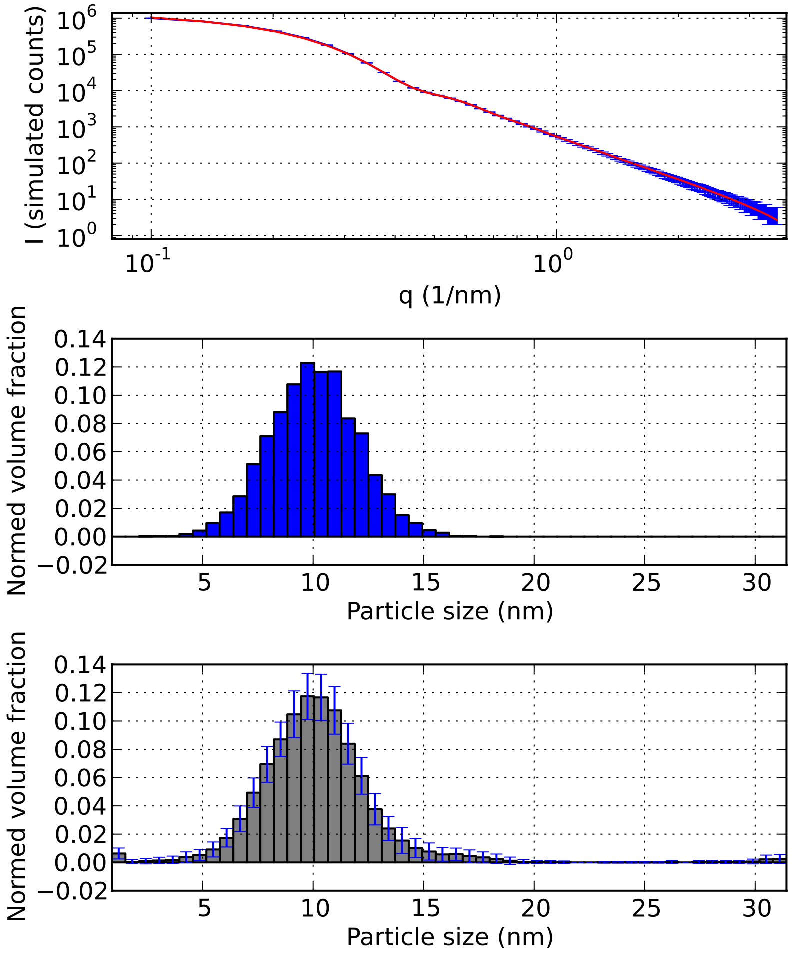

Fig. 2. Top: Simulated intensity (blue) and MC fit (red) of a narrow normal distribution of spheres with a mean of 10 nm and a width of 2 nm (middle). Bottom: Retrieved size distribution.

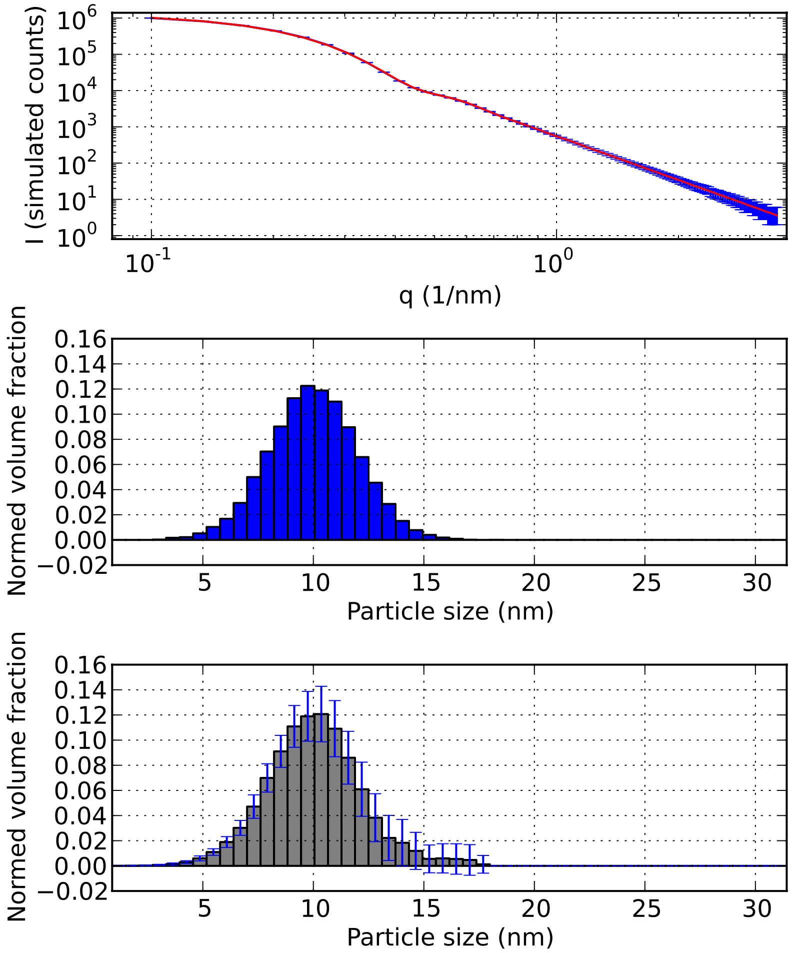

Fig. 3. Top: Simulated intensity (blue) and MC fit (red) of a narrow normal distribution of spheres with a mean of 10 nm and a width of 2 nm (middle), with $p_c = 6$. Bottom: Retrieved size distribution.

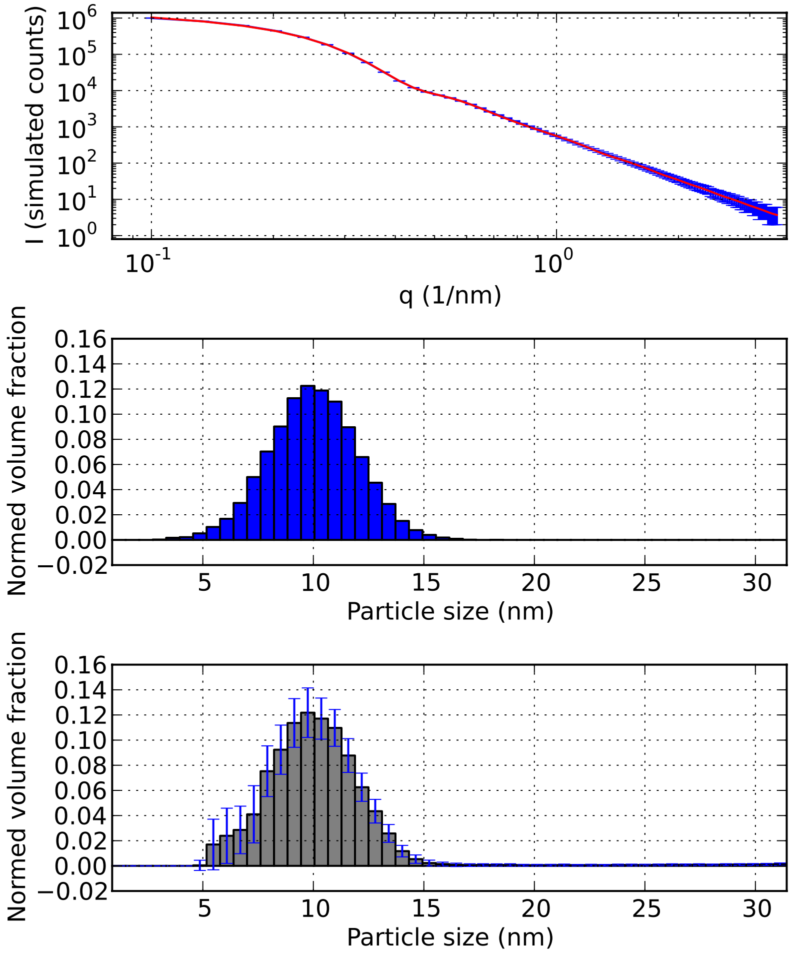

Fig. 4. Top: Simulated intensity (blue) and MC fit (red) of a narrow normal distribution of spheres with a mean of 10 nm and a width of 2 nm (middle), with $p_c = 0$. Bottom: Retrieved size distribution.

3.3. Wide normal distribution

A normal distribution with the mean at 10 nm and a standard deviation of 10 nm is simulated. The MC method has no problem retrieving this distribution (Figure 5). The uncertainty becomes larger with wide distributions.

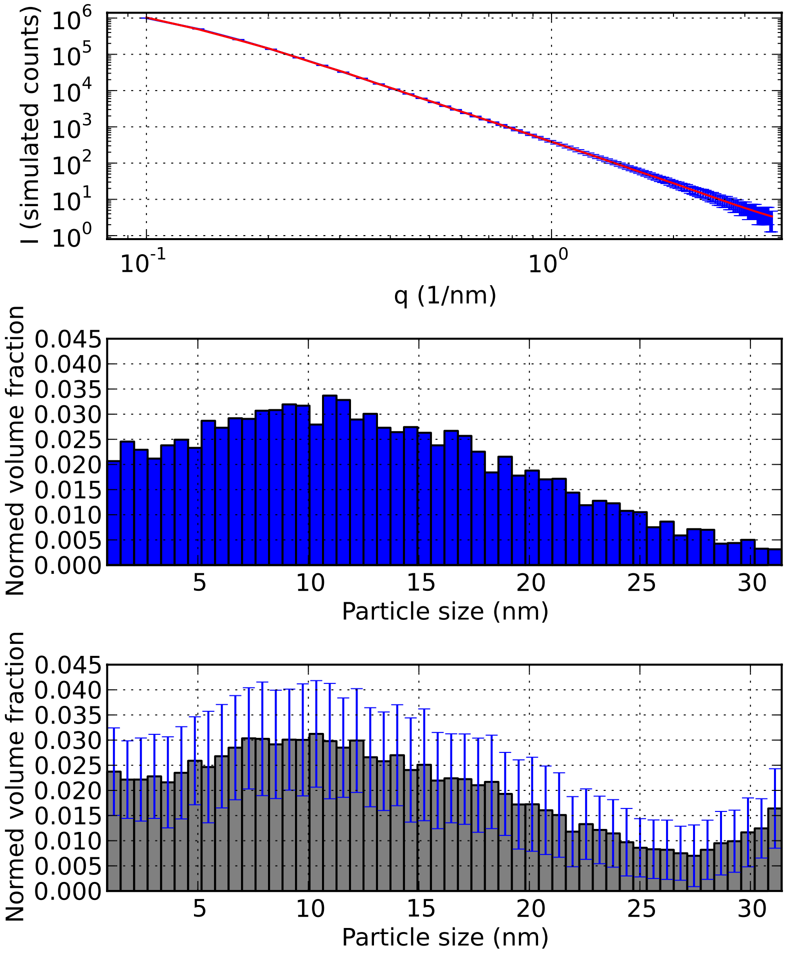

Fig. 5. Top: Simulated intensity (blue) and MC fit (red) of a wide normal distribution of spheres with a mean of 10 nm and a width of 10 nm (middle). Bottom: Retrieved size distribution.

3.4. Uniform distribution

A uniform distribution is simulated within the bounds set by the q limits (i.e. between 0.9 and 32 nm). The MC method has no problem retrieving this, but the uncertainties on the distributions becomes very large (Figure 6).

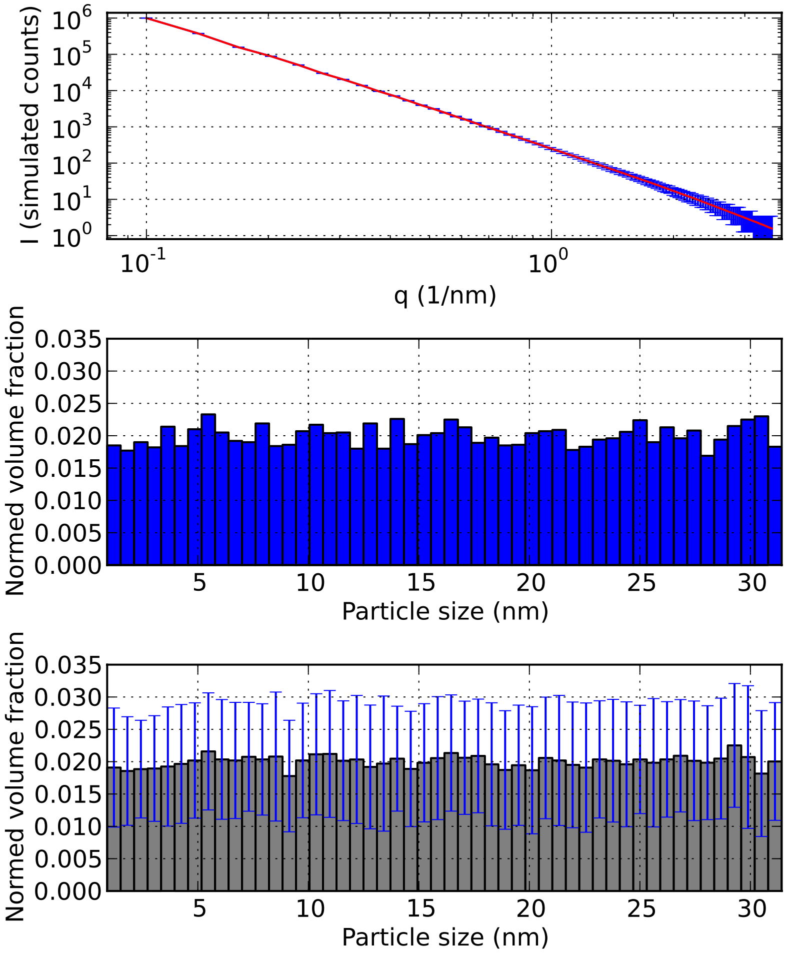

Fig. 6. Top: Simulated intensity (blue) and MC fit (red) of a uniform distribution of spheres ranging from 0.9 to 32 nm (middle). Bottom: Retrieved size distribution.

The uncertainties are an estimate, and are affected by the number of Monte-Carlo spheres used in the optimisation (c.f. Figure 7). Using a much larger number of spheres in the Monte-Carlo method does reduce the uncertainty for a much smoother solution, but it is considered wise to keep the number of spheres small as not to overly affect the result. The method here might be to determine the absolute minimum number of MC spheres to arrive at a (very coarse) solution, and then to multiply that number by a factor of 10-20 to arrive at a smooth solution.

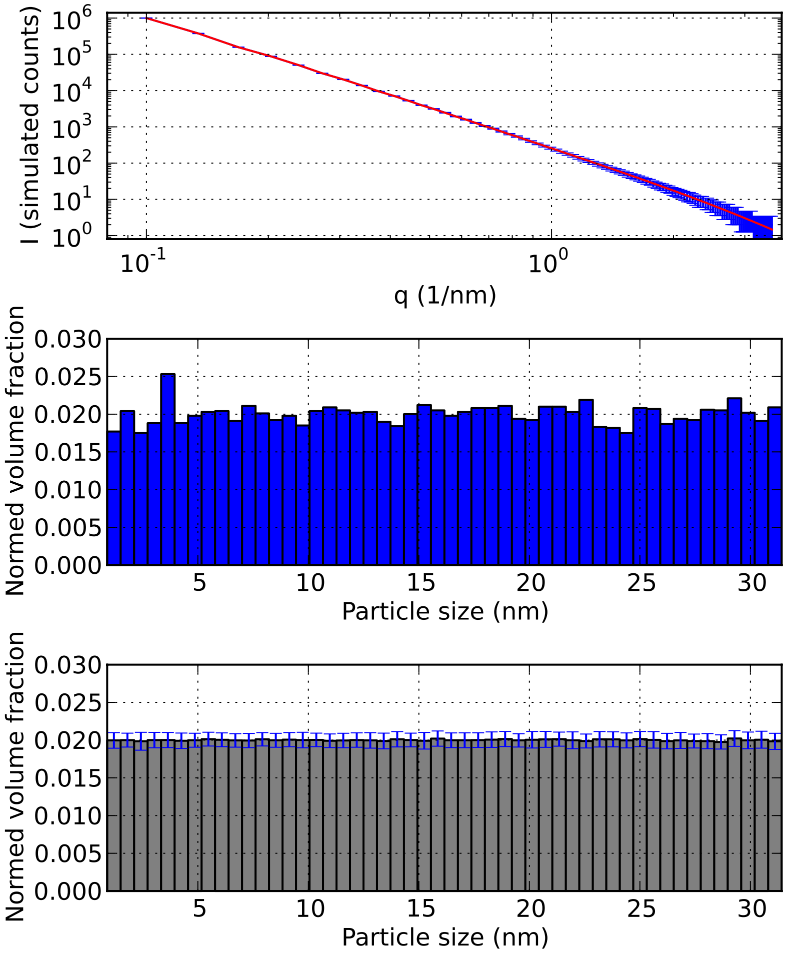

Fig. 7. Top: Simulated intensity (blue) and MC fit using 20000 MC spheres (red) of a uniform distribution of spheres ranging from 0.9 to 32 nm (middle). Bottom: Retrieved size distribution.

3.5. Triangular distributions

The scattering of three triangular distributions are calculated with:

1. A lower limit of 5nm, a maximum at 15nm and an upper limit of 15 nm (Figure 8),
2. A lower limit of 5nm, a maximum at 10nm and an upper limit of 15 nm (Figure 9),

3. A lower limit of 5nm, a maximum at 5nm and an upper limit of 15 nm (Figure 10),

The MC method has no problem retrieving these distributions, though the sharp edges lose some of their resolution at large sizes.

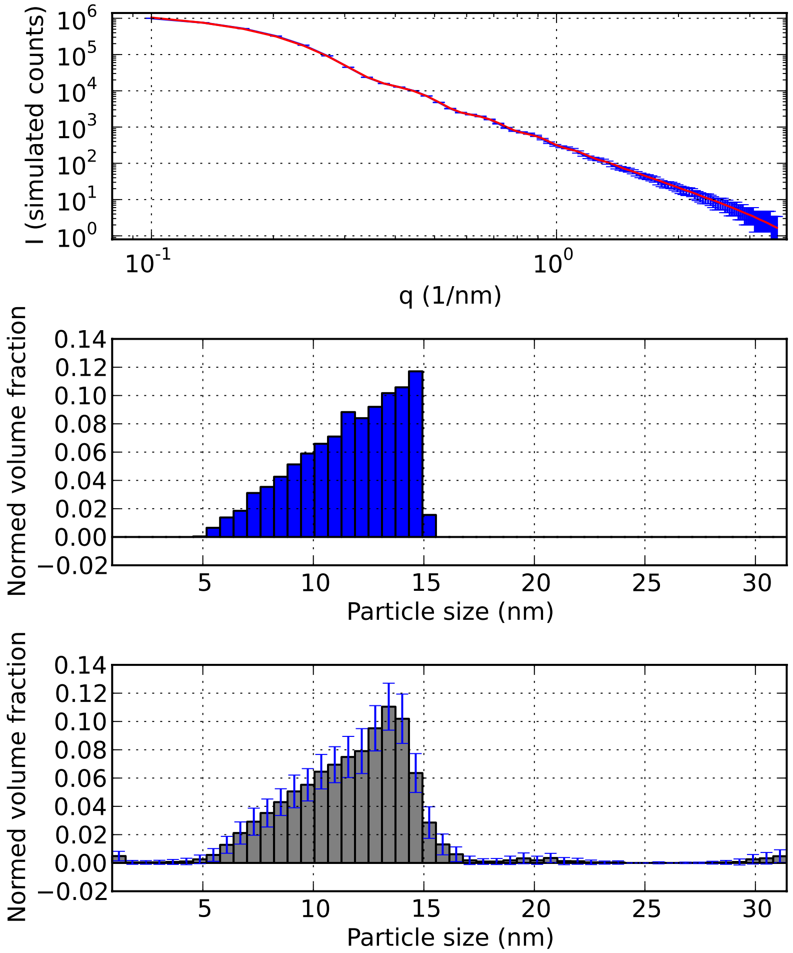

Fig. 8. Top: Simulated intensity (blue) and MC fit (red) of a triangular distribution with a lower limit of 5 nm, a maximum at 15 nm and an upper limit at 15 nm. Bottom: Retrieved size distribution.

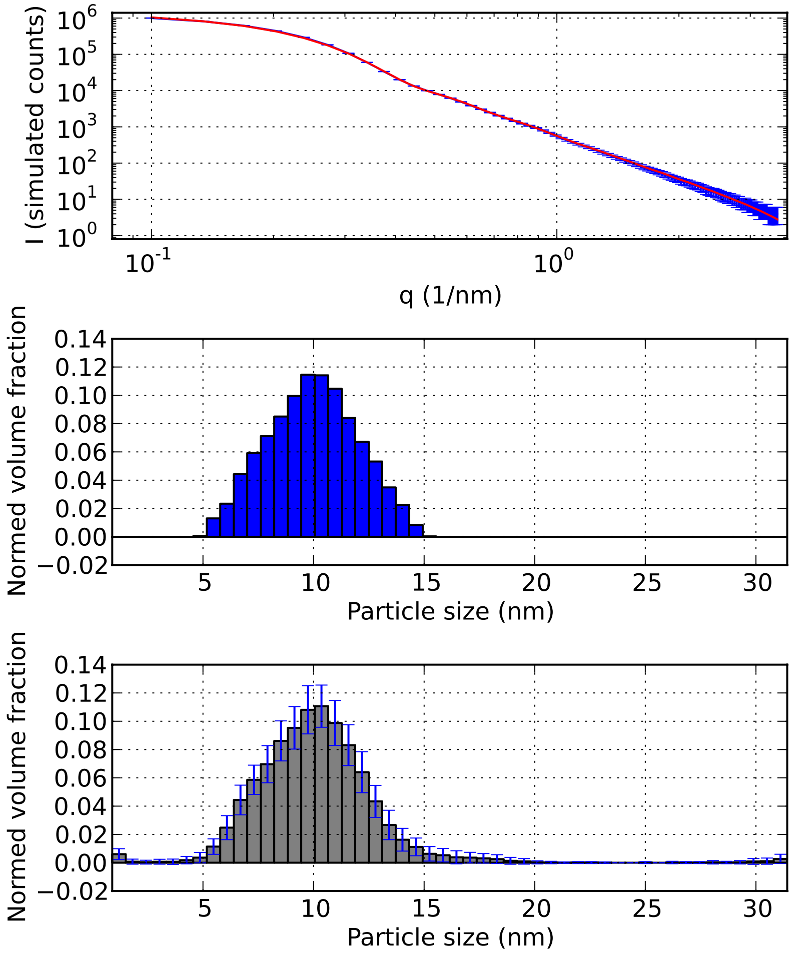

Fig. 9. Top: Simulated intensity (blue) and MC fit (red) of a triangular distribution with a lower limit of 5 nm, a maximum at 10 nm and an upper limit at 15 nm. Bottom: Retrieved size distribution.

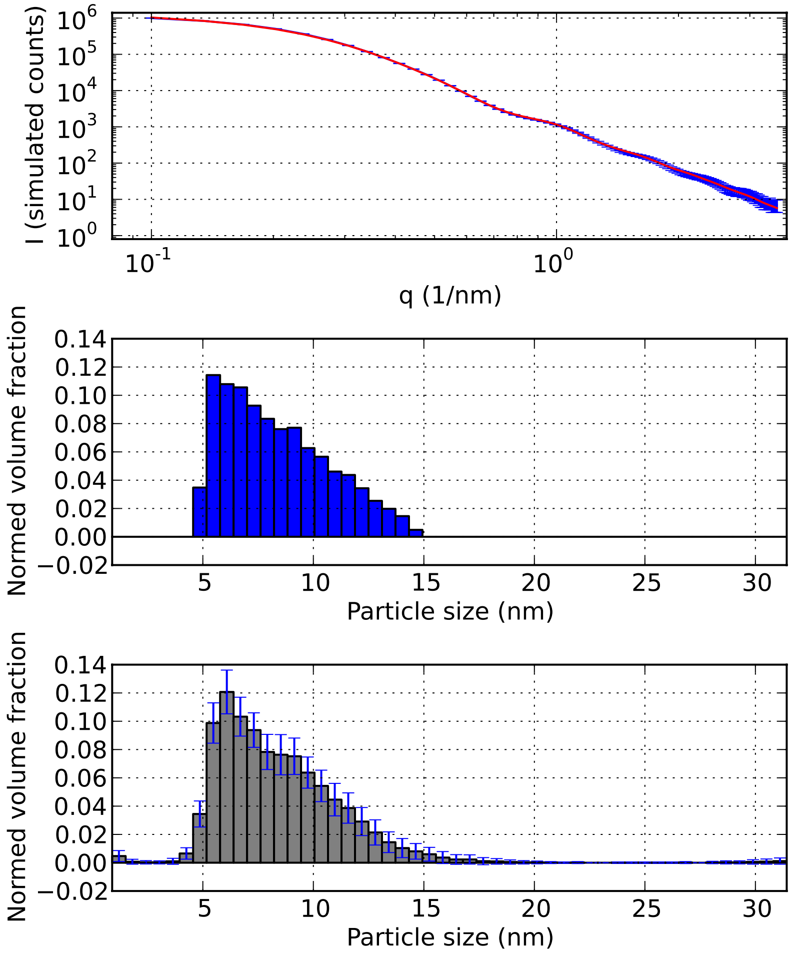

Fig. 10. Top: Simulated intensity (blue) and MC fit (red) of a triangular distribution with a lower limit of 5 nm, a maximum at 5 nm and an upper limit at 15 nm. Bottom: Retrieved size distribution.

4. Multimodal distributions

4.1. Bimodal normal distributions

Two normal distributions of spheres are combined, with means of 5 and 15 nm, and standard deviations of 2 and 2 nm. The MC method has no problem retrieving these (Figure 11).

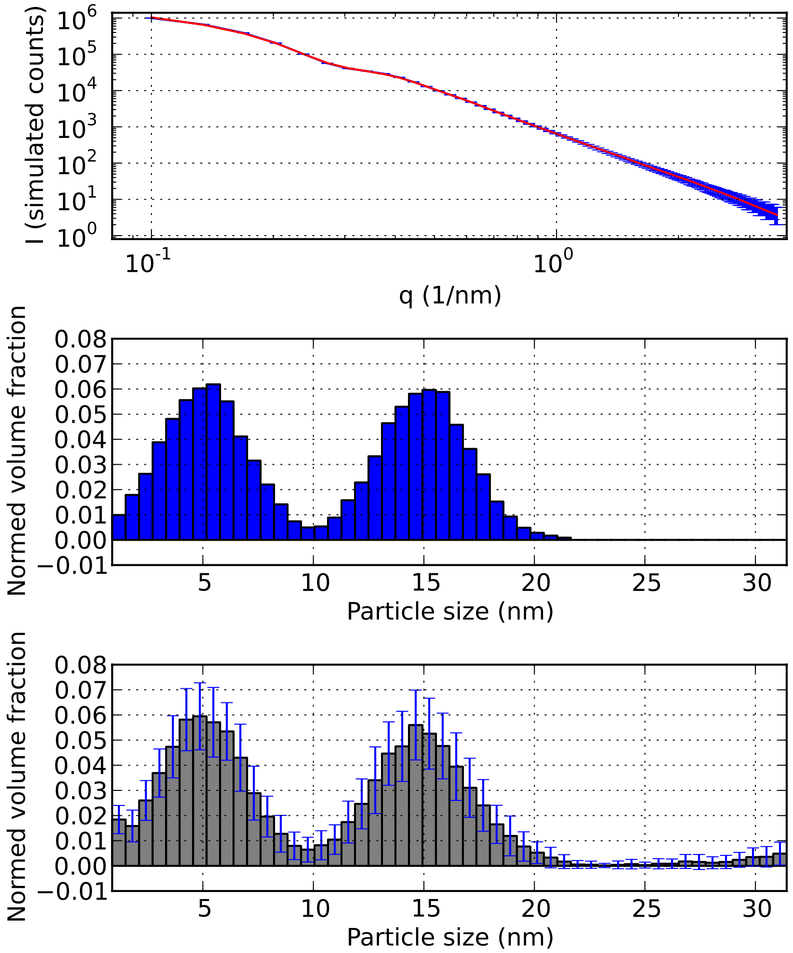

Fig. 11. Top: Simulated intensity (blue) and MC fit (red) of a combination of two normal distributions of spheres, with means of 5 and 15 nm, and standard deviations of 2 nm. (middle). Bottom: Retrieved size distribution.

4.2. Trimodal normal distributions

Three normal distributions of spheres are combined, with means of 5, 10 and 20 nm, and standard deviations of 2, 0.5 and 2 nm. The MC method has no problem retrieving these (Figure 12).

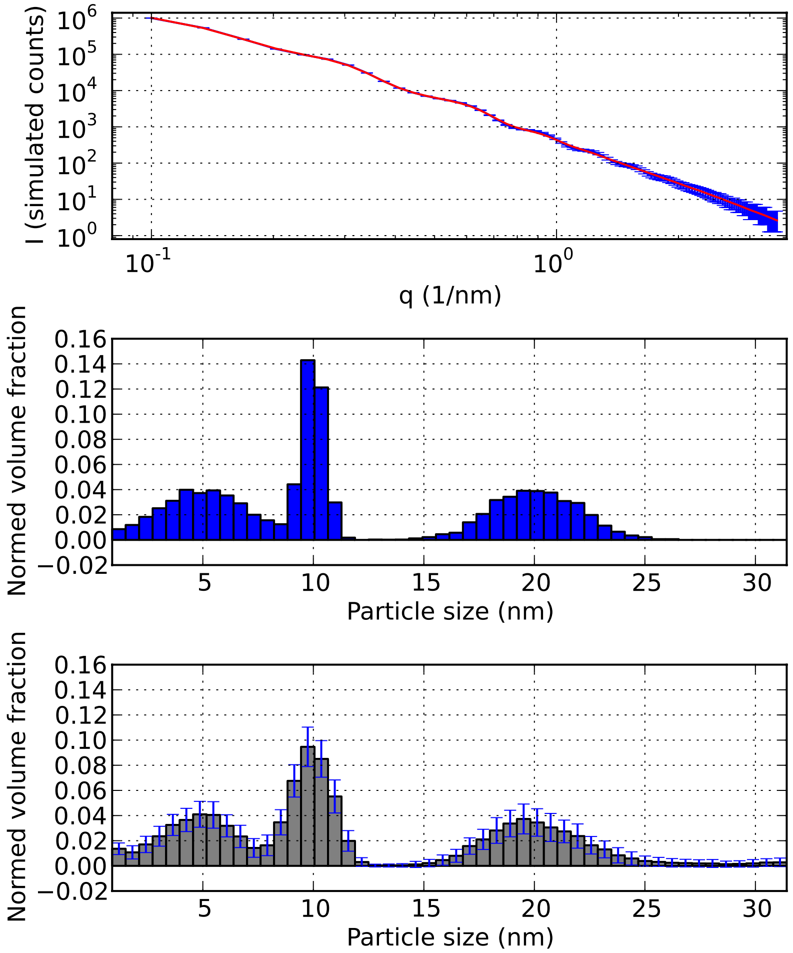

Fig. 12. Top: Simulated intensity (blue) and MC fit (red) of a combination of three normal distributions of spheres, with means of 5, 10 and 20 nm, and standard deviations of 2, 0.5 and 2 nm. (middle). Bottom: Retrieved size distribution.

4.3. Quadmodal normal distributions

Four normal distributions of spheres are combined, with means of 5, 10, 15 and 25 nm, and standard deviations of 2, 0.5, 2 and 0.5 nm. The MC method has no problem retrieving these, although the sharpness of the last size distribution is reduced (Figure 13).

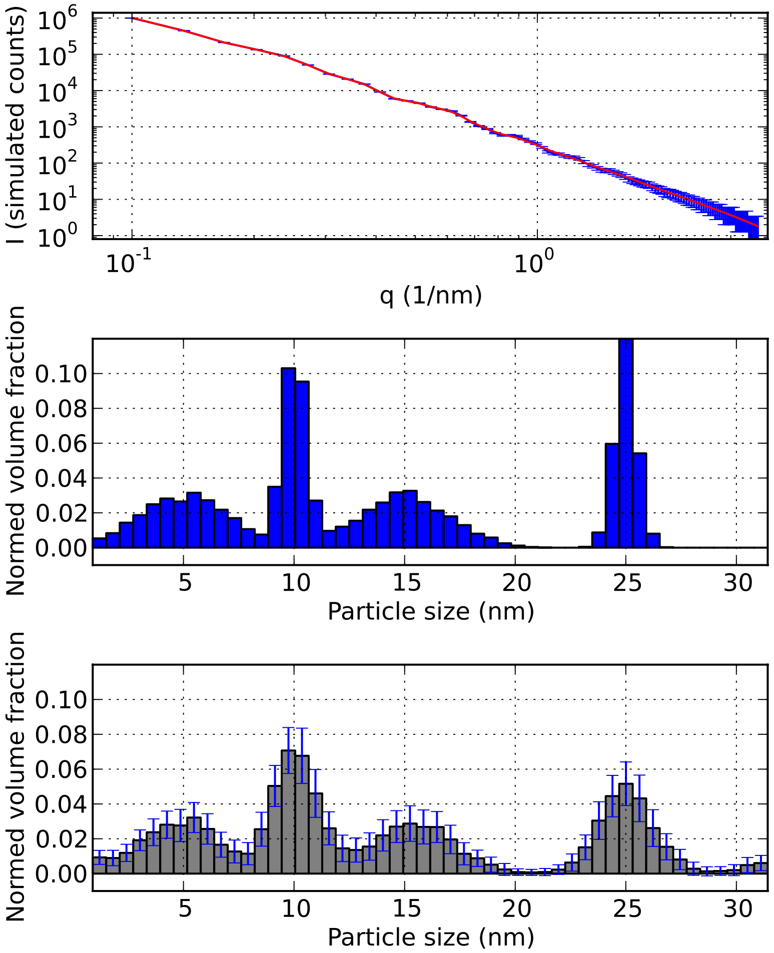

Fig. 13. Top: Simulated intensity (blue) and MC fit (red) of a combination of four normal distributions of spheres, with means of 5, 10, 15 and 25 nm, and standard deviations of 2, 0.5, 2 and 0.5 nm. (middle). Bottom: Retrieved size distribution.

4.4. Multimodal monodisperse distributions

Five monodisperse sets of spheres are combined, with sizes of 2, 4, 7, 14 and 25 nm. The MC method has no problem retrieving these, although it appears that the resolution of the size retrieval of the larger sizes decreases (Figure 14).

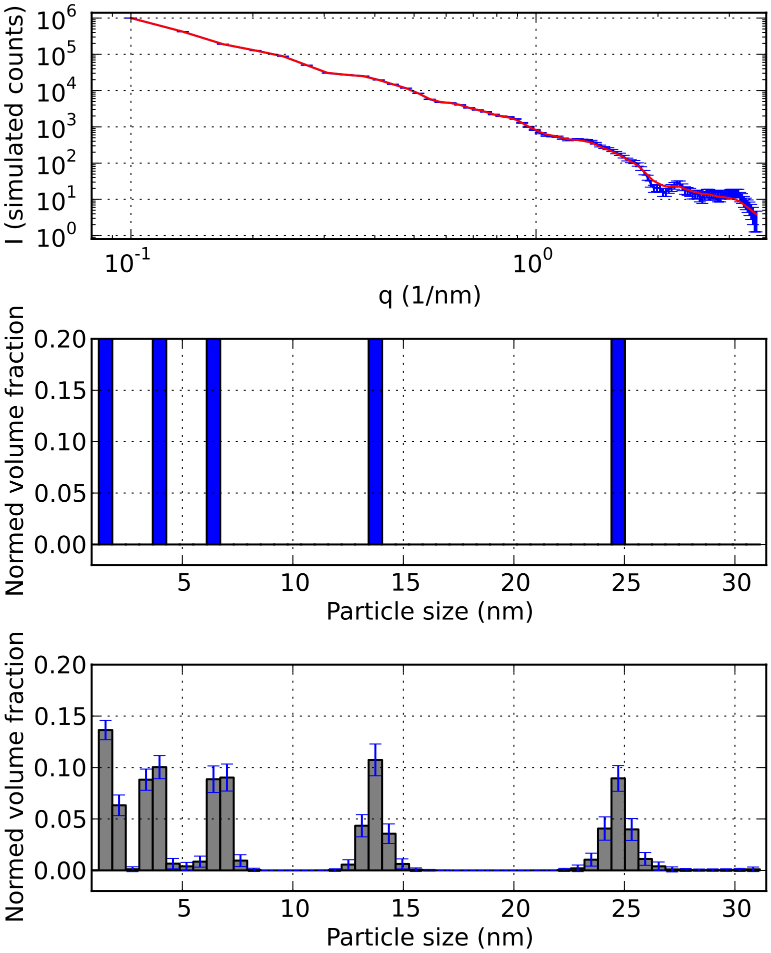

Fig. 14. Top: Simulated intensity (blue) and MC fit (red) of a multimodal set of monodisperse spheres with sizes of 2, 4, 7, 14 and 25 nm (middle). Bottom: Retrieved size distribution.

It is possible that this reduction in resolution is related to the level (and therefore accuracy) of the intensity, so we retry the fit using a 100-fold increase in simulated counts. Indeed, this appears to retrieve the high end of the distributions better (Figure 15).

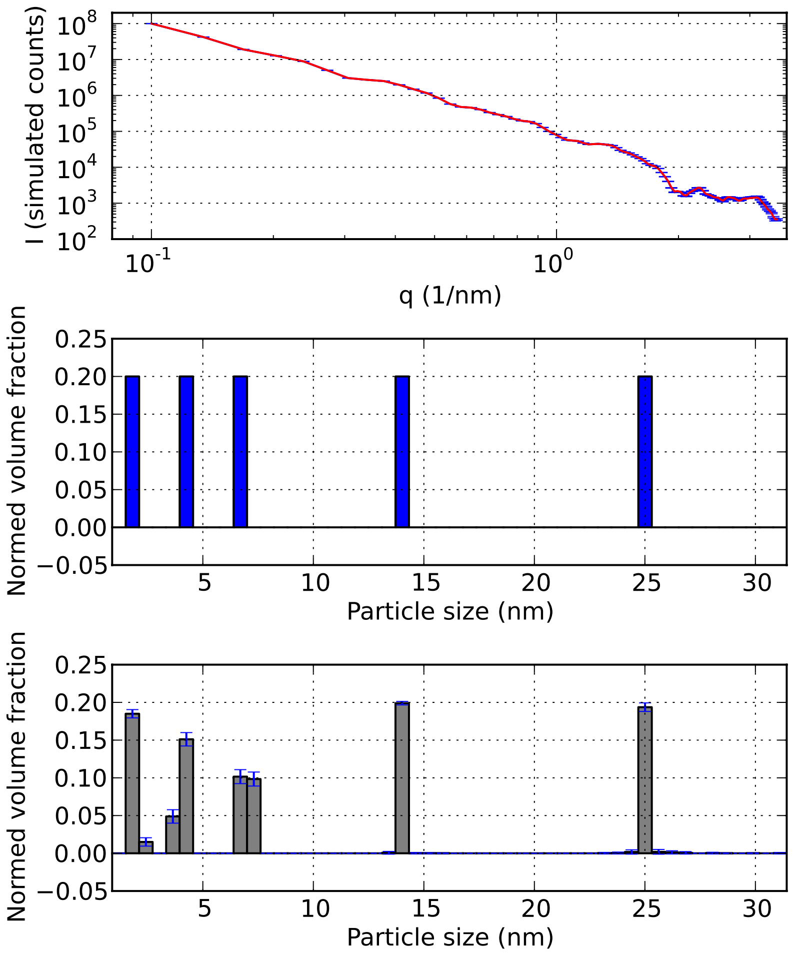

Fig. 15. Top: High level of simulated intensity (blue) and MC fit (red) of a multimodal set of monodisperse spheres with sizes of 2, 4, 7, 14 and 25 nm (middle). Bottom: Retrieved size distribution.

4.5. Mixed triangular and large monodisperse

A triangular distribution of spheres ranging from 5 to 15 nm with its maximum at 5 is combined with a monodisperse set of spheres of 20 nm. The MC method has no problem retrieving this, though the monodisperse set has lost its sharpness (probably due to a lack of intensity as shown for the multimodal monodisperse distribution, Figure 16).

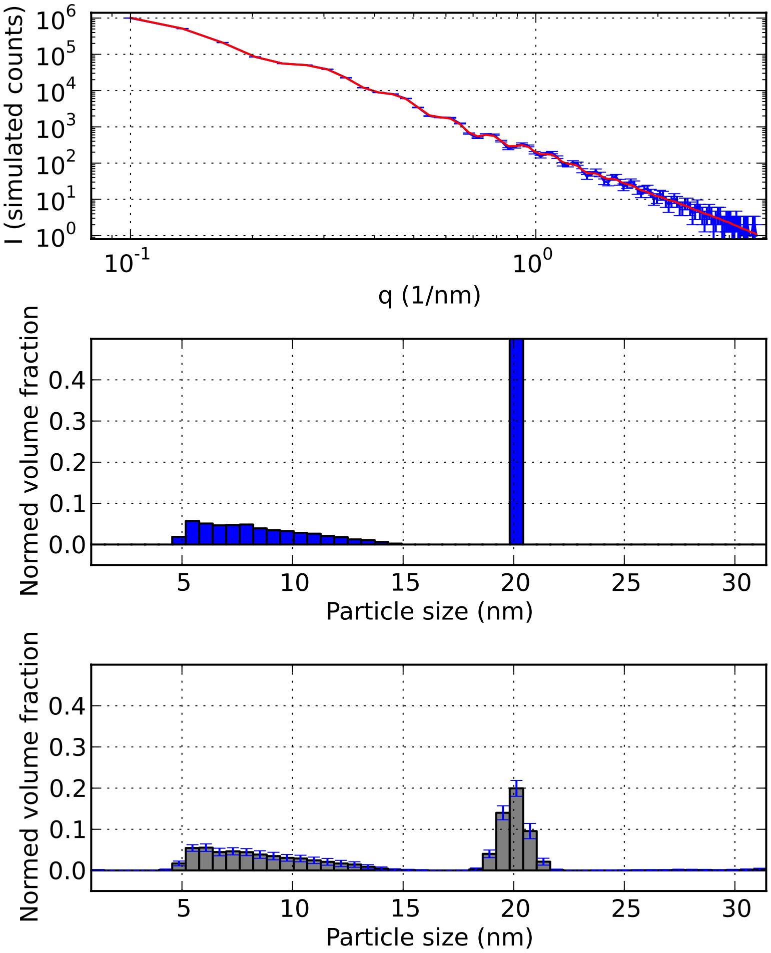

Fig. 16. Top: Simulated intensity (blue) and MC fit (red) of a triangular distribution of spheres (5-15 nm with a maximum at 5 nm) combined with a monodisperse set of 20 nm spheres (middle). Bottom: Retrieved size distribution.

Similarly, a set of 5 nm spheres is added to a triangular distribution ranging from 10-20 nm, with a maximum at 10 nm. The MC method has no problems retrieving this (Figure 17).

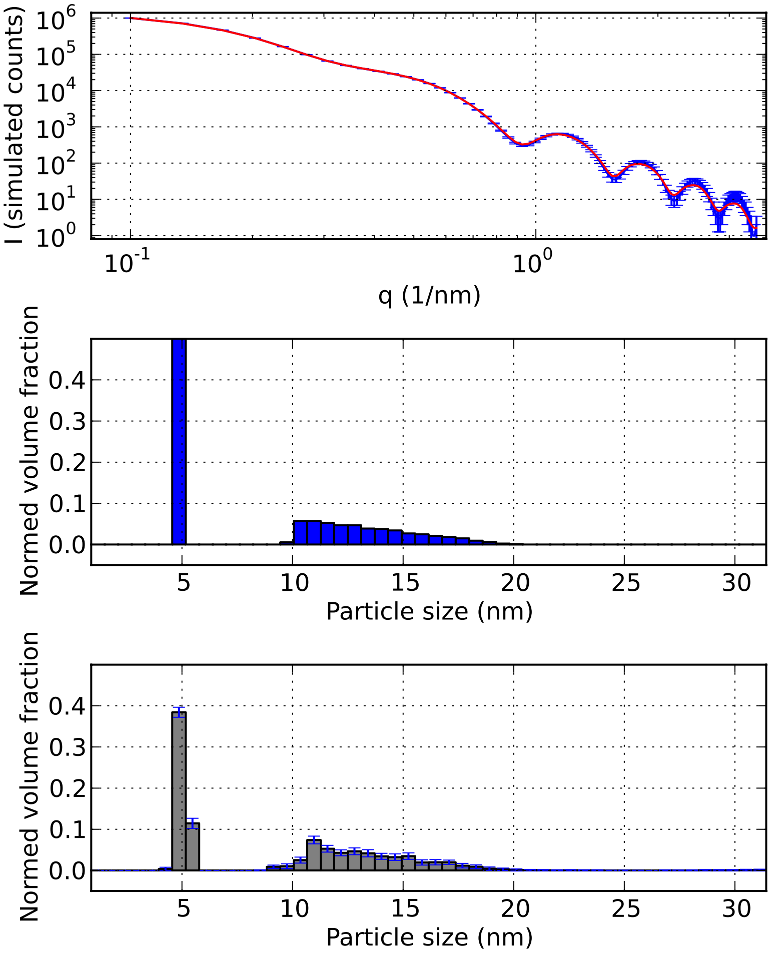

Fig. 17. Top: Simulated intensity (blue) and MC fit (red) of a triangular distribution of spheres (10-20 nm with a maximum at 10 nm) combined with a monodisperse set of 5 nm spheres (middle). Bottom: Retrieved size distribution.

5. Distributions exceeding the upper size bounds

5.1. Multimodal monodisperse spheres

In order to investigate the upper limits of size distribution retrieval, The scattering from five monodisperse sets of spheres are combined, with sizes of 14, 25, 35, 50 and 70 nm. The Monte-Carlo size limits for this are set to 0.9 and 100 nm. The rule-of-thumb that the maximum distinguishable radius R_{max} is linked to the minimum

measured q through $R_{max} = \pi/q_{min}$ indicates that we should not be able to distinguish anything with radii above 32 nm. When supplied with the standard parameters, there are spurious peaks appearing beyond 32 nm, possibly harmonics of some of the larger sizes (Figure 18).

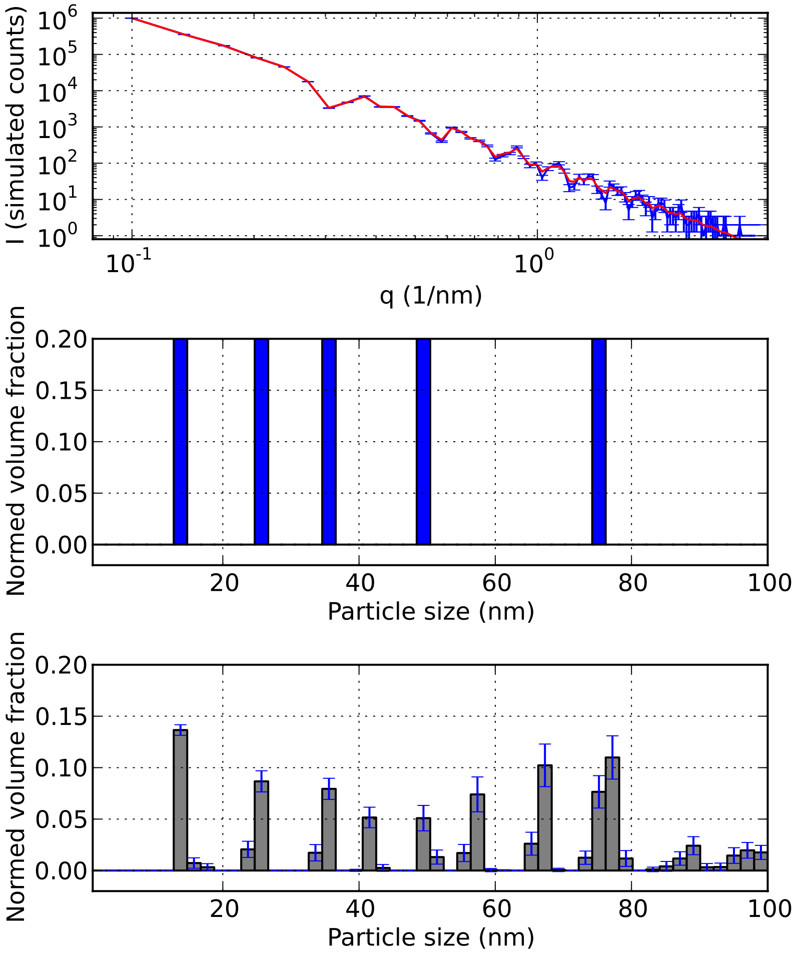

Fig. 18. Top: Simulated intensity (blue) and MC fit (red) of a multimodal set of monodisperse spheres with sizes of 14, 25, 35, 50 and 75 nm (middle). Bottom: Retrieved size distribution.

Figure 19 shows that when the intensity is sampled over 1000 as opposed to 100 points in q-space (and correspondingly lowering the intensity), most of the incorrect sizes are no longer present in the retrieved size distribution. This interesting result

shows that some care might be necessary when choosing the number of sampling points in q-space, and implies that the maximum determinable size is not simply related to the minimum measurable q. In line with the discrete Fourier transform methods, the maximum measurable size may perhaps also be related to the distance between the Fourier sampling points. Some closer investigation into this issue may be worthwhile, but is well beyond the scope of this paper.

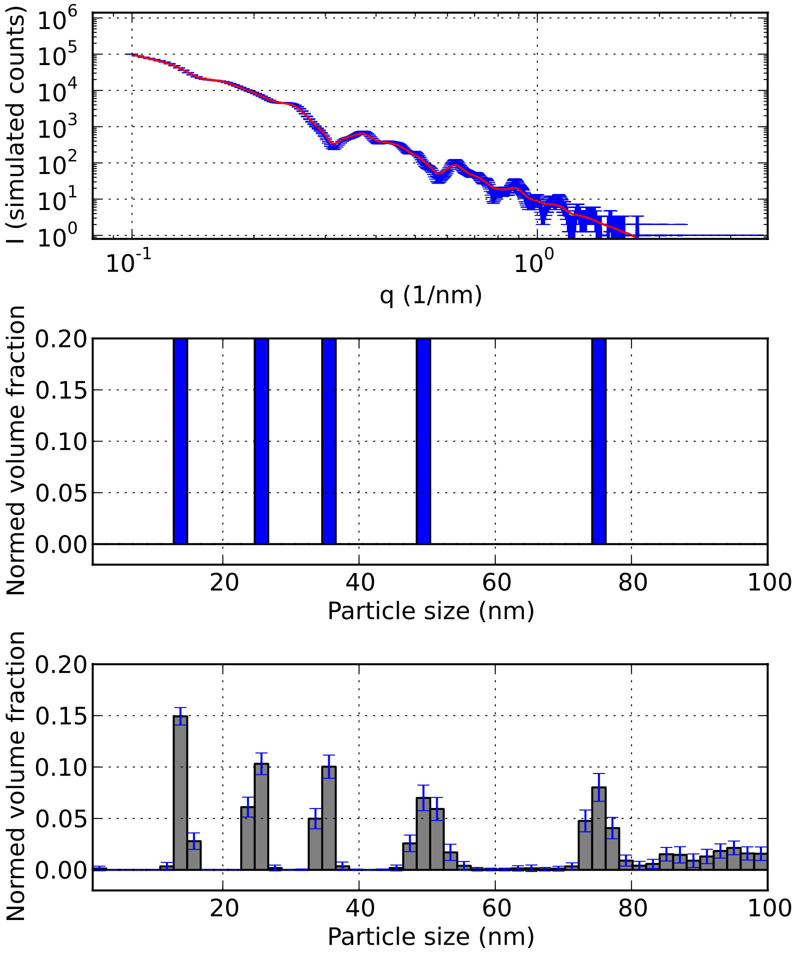

Fig. 19. Top: Simulated intensity (blue) sampled over 1000 q-points and MC fit (red) of a multimodal set of monodisperse spheres with sizes of 14, 25, 35, 50 and 75 nm (middle). Bottom: Retrieved size distribution.

6. Distributions exceeding the lower size bounds

That leaves the question on what happens when the lower size bounds are exceeded: can we determine the size of particles below π/q_{max}? To investigate this, the scattering from five monodisperse sets of spheres are combined, with sizes of 0.15, 0.35, 0.75, 1 and 2 nm. The MC solution shows a clear inability to accurately distinguish between these sizes (Figure 20). the Monte-Carlo fit size limits are set for this to 0.05 and 5 nm.

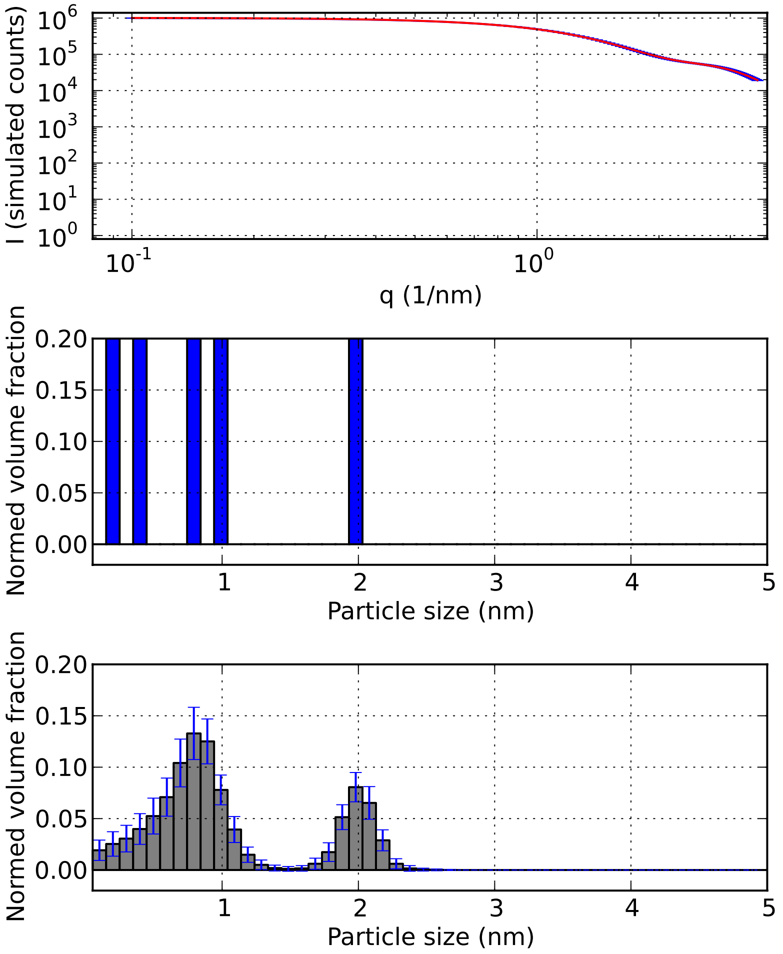

Fig. 20. Top: Simulated intensity (blue) and MC fit (red) of a multimodal set of monodisperse spheres with sizes of 0.15, 0.35, 0.75, 1 and 2 nm (middle). Bottom: Retrieved size distribution.

7. Distributions of other shapes

In the main paper describing the MC method, it is indicated that it is not possible in principle to uniquely separate the shape-information from size distribution information in small-angle scattering. Therefore, scattering from a distribution of non-spherical objects (or, as shown below, for at least prolate and oblate ellipsoids) can still be described using spherical objects, though the retrieved distribution is dissimilar from the original distribution.

The aspect ratio is defined here as the ratio between the meridional radius and the equatorial radius. The ellipsoid scattering is calculated for each sampled ellipsoid using the equations given in (Pedersen, 1997).

Two different aspect ratios have been simulated: 1) prolate and oblate ellipsoids with an aspect ratio of 2 and 0.5, respectively to investigate the effect of mild deviations on the distributions, and 2) prolate and oblate ellipsoids with an aspect ratio of 20 and 0.05, respectively. The latter has been added to highlight the aforementioned ability to fit scattering even from such extreme shapes to polydisperse spheres.

7.1. Monodisperse spherically isotropic ellipsoids with an aspect ratio of 2 and 0.5

A monodisperse set of ellipsoids with an equatorial radius of 10 nm and a meridional radius of 20 nm is simulated (i.e. an aspect ratio of 2). The MC method has no problem fitting the scattering assuming spheres, but the retrieved size distribution is skewed to larger sizes (Figure 21). For oblate ellipsoids with an aspect ratio of $\frac{1}{2}$ and an equatorial radius of 10 nm, the intensity can be equally fit, and the retrieved size distribution is skewed to smaller sizes (Figure 22)

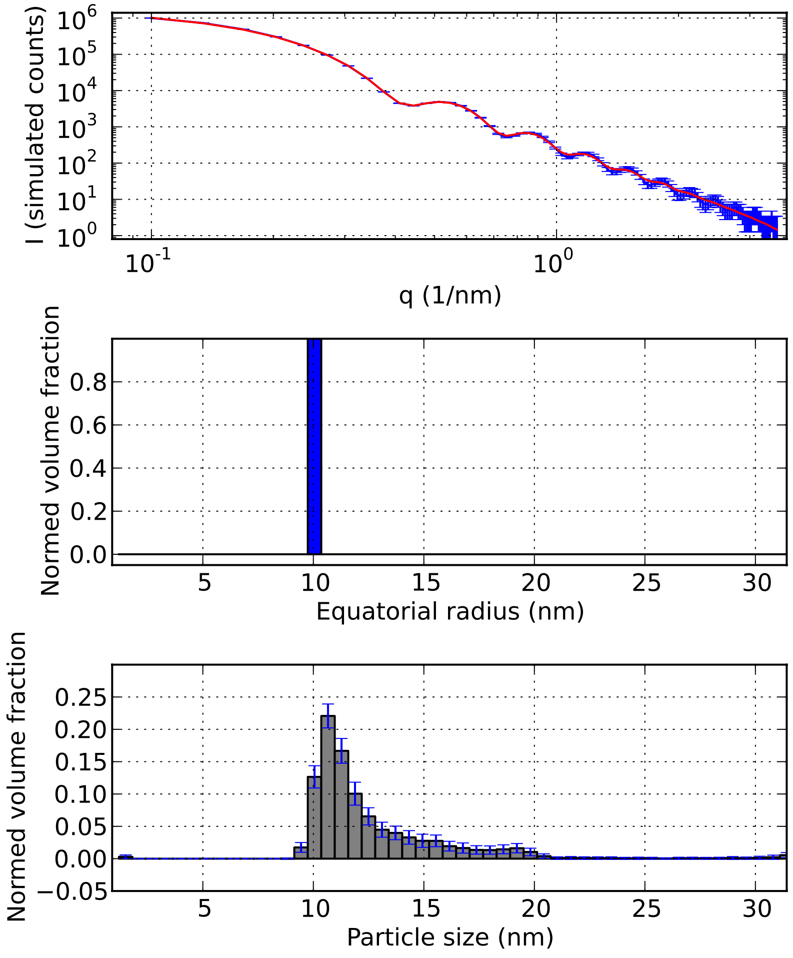

Fig. 21. Top: Simulated intensity (blue) and MC fit (red) of a monodisperse distribution of prolate ellipsoids with an equatorial radius of 10 nm and meridional radius of 20 nm (middle). Bottom: Retrieved size distribution.

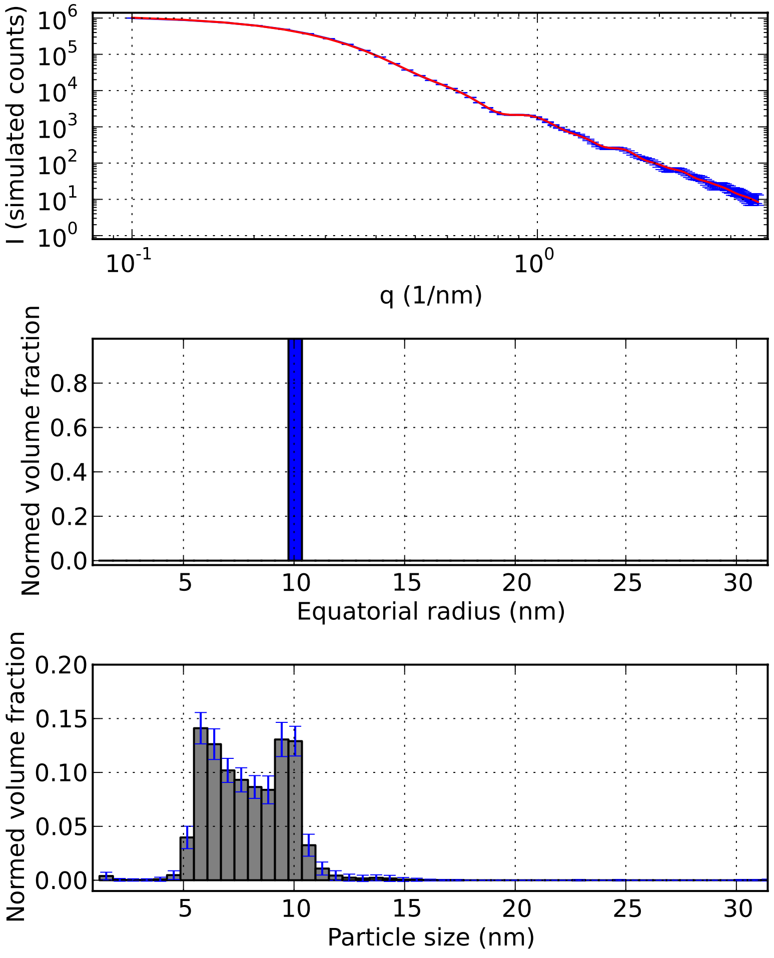

Fig. 22. Top: Simulated intensity (blue) and MC fit (red) of a monodisperse distribution of oblate ellipsoids with an equatorial radius of 10 nm and meridional radius of 5 nm (middle). Bottom: Retrieved size distribution.

7.2. polydisperse spherically isotropic ellipsoids with an aspect ratio of 2 and 0.5

A set of prolate ellipsoids (aspect ratio of 2) with equatorial radii sampled over a normal size distribution with a mean of 10 nm and a standard deviation of 2 nm is simulated. The MC method has no problem fitting the scattering assuming spheres, but the retrieved size distribution is skewed to larger sizes (Figure 23). For oblate ellipsoids with the same equatorial radius distribution but an aspect ratio of $\frac{1}{2}$, the

intensity can be equally fit, and the retrieved size distribution is skewed to smaller sizes (Figure 24)

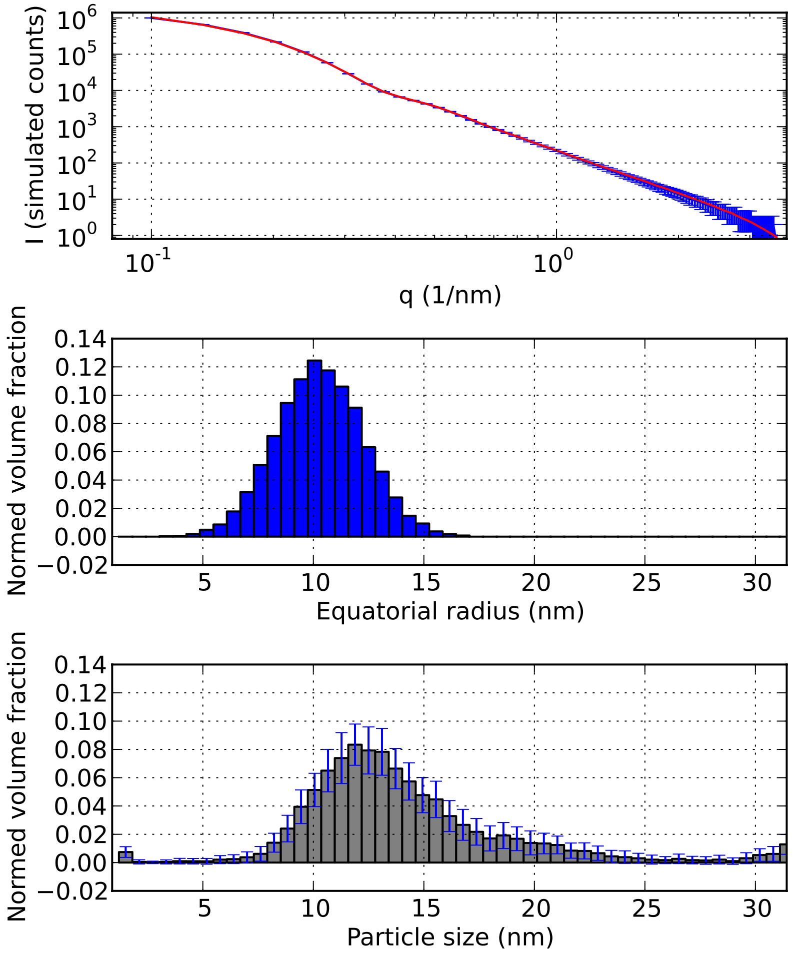

Fig. 23. Top: Simulated intensity (blue) and MC fit (red) of prolate ellipsoids with an equatorial radius normally distributed with a mean of 10 nm and standard deviation of 2 nm. The aspect ratio is 2. Bottom: Retrieved size distribution.

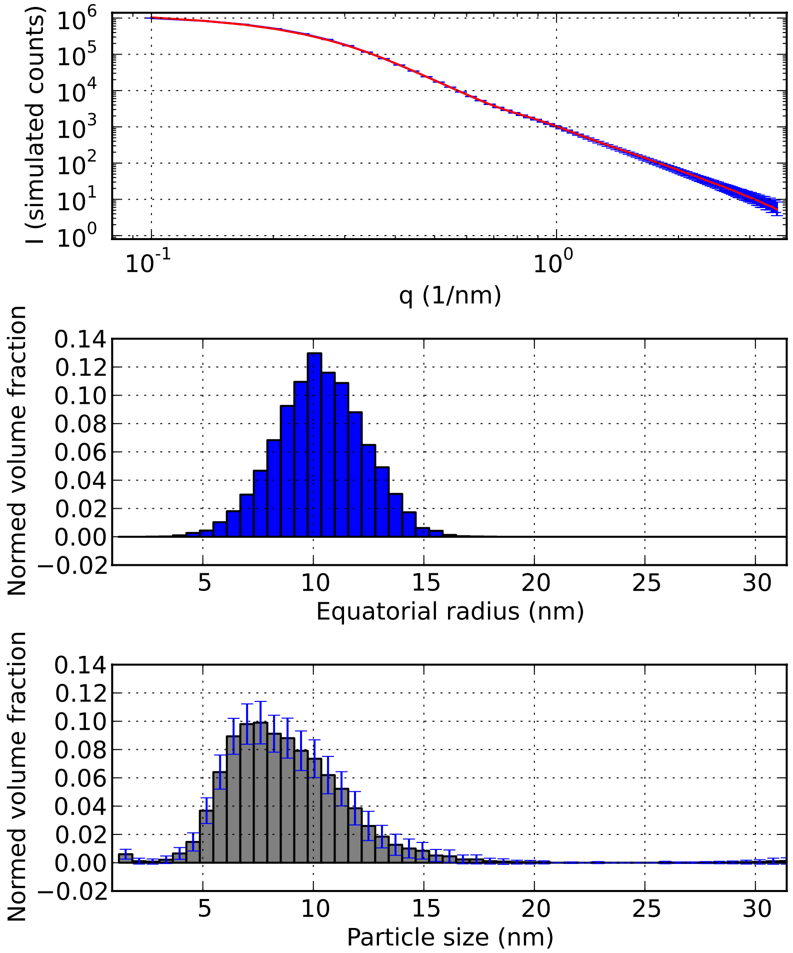

Fig. 24. Top: Simulated intensity (blue) and MC fit (red) of oblate ellipsoids with an equatorial radius normally distributed with a mean of 10 nm and standard deviation of 2 nm. The aspect ratio is $\frac{1}{2}$. Bottom: Retrieved size distribution.

7.3. Monodisperse spherically isotropic ellipsoids with an aspect ratio of 20 and 0.05

A monodisperse set of ellipsoids with an equatorial radius of 10 nm and a meridional radius of 200 nm is simulated (i.e. an aspect ratio of 20). The MC method has no problem fitting the scattering assuming spheres, but the retrieved size distribution is skewed to larger sizes (Figure 25). For oblate ellipsoids with an aspect ratio of $\frac{1}{20}$ and an equatorial radius of 10 nm, the intensity can be equally fit, and the retrieved size

distribution is skewed to smaller sizes (Figure 26)

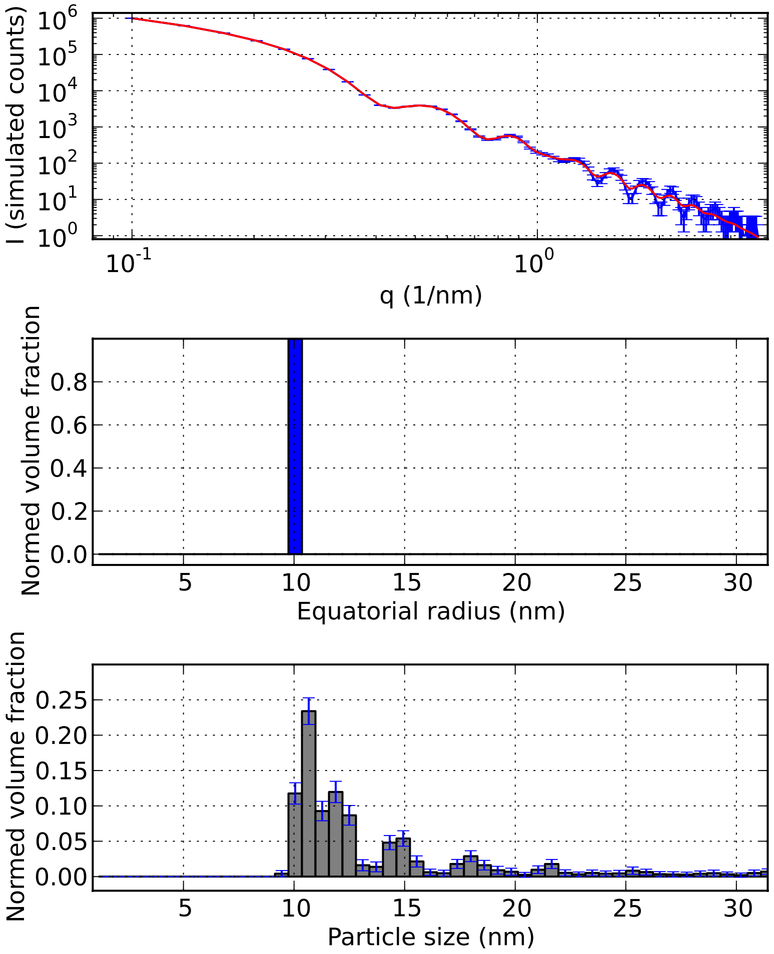

Fig. 25. Top: Simulated intensity (blue) and MC fit (red) of a monodisperse distribution of prolate ellipsoids with an equatorial radius of 10 nm and meridional radius of 200 nm (middle). Bottom: Retrieved size distribution.

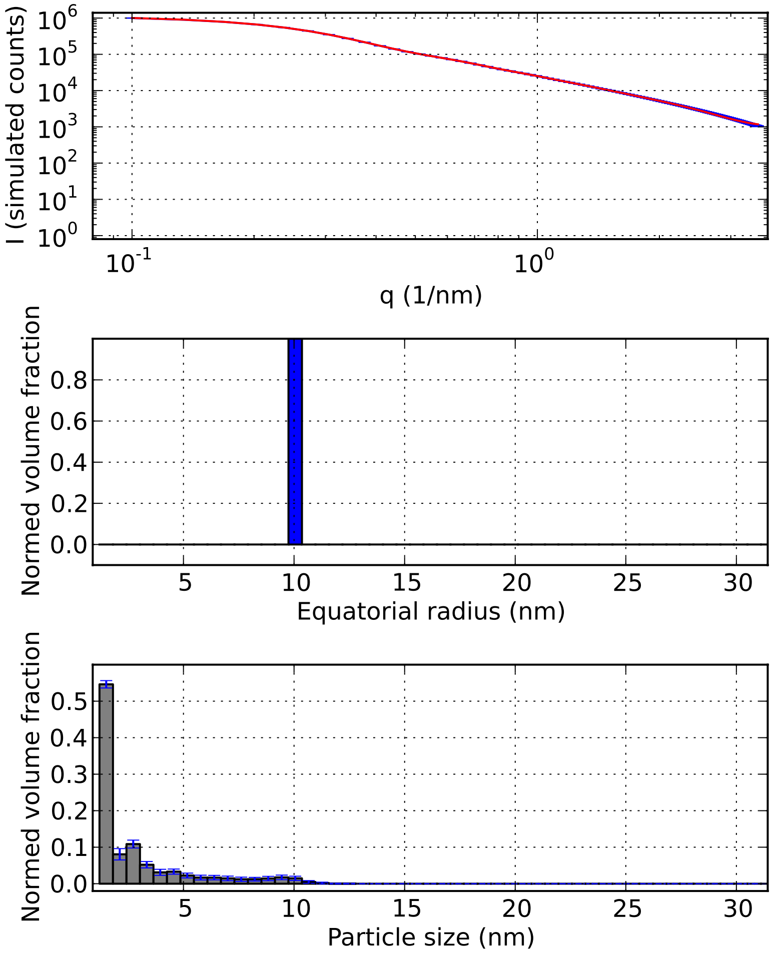

Fig. 26. Top: Simulated intensity (blue) and MC fit (red) of a monodisperse distribution of oblate ellipsoids with an equatorial radius of 10 nm and meridional radius of 0.5 nm (middle). Bottom: Retrieved size distribution.

It stands to reason that, since the monodisperse ellipsoids with aspect ratios of 20 and 0.05 can be described using spheres, polydisperse sets of these will similarly be describable. The resulting size distributions will be skewed as above.

8. Python details:

All graphs were calculated using Python 2.7 and iPython 0.11, in particular:

```
Python 2.7.2 |EPD 7.1-2 (32-bit)| (default, Jul 27 2011, 13:29:32)
```

the latest version of saxstools.py and picklefunctions.py code containing the scattering pattern generation and MC analysis functions can be freely obtained from the author.

References

Pedersen, J. S. (1997). *Adv. Coll. Interf. Sci.* **70**, 171–210.
